# Wettability-Tuned Polydopamine-Modified Surfaces for Enhanced Dropwise Condensation and Water Harvesting

**DOI:** 10.1021/acs.langmuir.6c04242

**Published:** 2026-10-15

**Authors:** Nai-Ci Zheng, Bo-Yan Huang, Li-Jen Chen

**Affiliations:** Department of Chemical Engineering, 33561 National Taiwan University, Taipei 10617, Taiwan

## Abstract

Low-surface-energy superhydrophobic surfaces facilitate droplet removal but often exhibit a high nucleation energy barrier, hindering water-harvesting efficiency. To address this limitation, appropriately increasing surface hydrophilicity offers a promising strategy to enhance droplet nucleation while preserving sufficient droplet mobility. In this study, the surface wettability of SiO_2_ nanoparticle-textured superhydrophobic substrates (SHB) was systematically regulated by controlling the polydopamine (PDA) reaction time. Increasing the PDA reaction time gradually enhanced surface hydrophilicity, resulting in asynchronous transitions in sessile-droplet wetting and condensation mode. The Cassie-to-Wenzel transition of sessile droplets occurred at a PDA reaction time of 9 min. In contrast, the transition from dropwise condensation (DWC) to filmwise condensation (FWC) was delayed until a PDA reaction time of 11 min. The PDA10 substrate (the surface prepared at a PDA reaction time of 10 min) exhibited the optimal condensation performance, achieving enhanced droplet nucleation without excessive loss of droplet mobility. Compared with the PDA0 (SHB) substrate, the PDA10 substrate increased the condensate mass collection rate by 62.0% and the heat transfer coefficient by 61.4%. These findings demonstrate that temporal control of PDA modification enables regulation of distinct wetting and condensation transition thresholds, providing a design strategy to improve condensation-based water harvesting and heat transfer.

## Graphical abstract

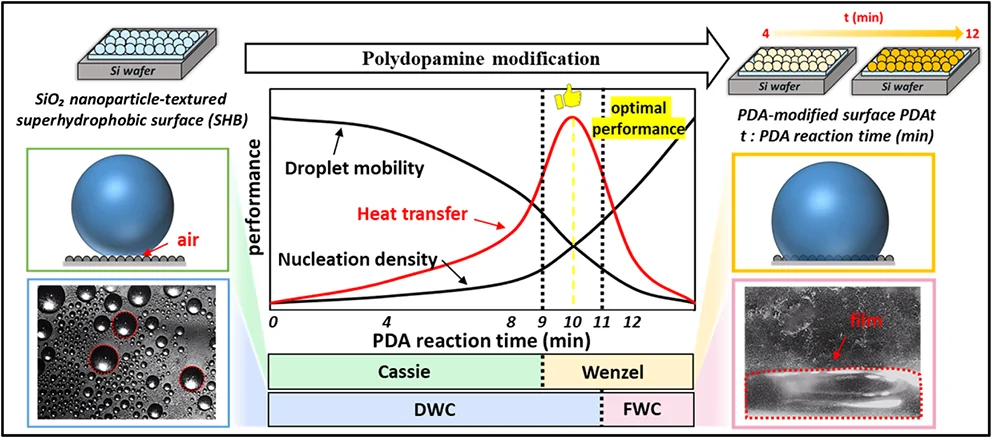

## Introduction

Condensation is a common natural phenomenon and an indispensable process in many industrial applications, such as steam condensers used in steam power plants.^1^ Vapor condensation occurs in two distinct modes: dropwise condensation (DWC) and filmwise condensation (FWC), governed by surface hydrophobicity and hydrophilicity, respectively. DWC can increase heat-transfer performance by at least an order of magnitude compared with FWC, thereby reducing the high thermal resistance associated with water-film accumulation.^2–4^ Drawing inspiration from nature, the Namib desert beetle has been widely studied for its high-efficiency water-harvesting capability, which is closely associated with the regulation of surface wettability.^5,6^ Such surfaces have been applied in various applications, including water collection,^7–9^ heat transfer,^10–13^ and anti-icing.^14,15^

The fundamental wetting behaviors on rough and heterogeneous substrates are traditionally well described by the Wenzel^16^ and Cassie–Baxter^17^ models, respectively. In the Cassie state, trapped air pockets within the surface microstructures prevent liquid infiltration, whereas the Wenzel state features completely wetted surface grooves with no air pockets. This structural wetting behavior directly influences phase-change performance. During condensation, hydrophilic surfaces efficiently capture atmospheric moisture due to a lower nucleation energy barrier. Still, their strong adhesion makes it highly challenging to remove the resulting Wenzel-like water film. Conversely, hydrophobic surfaces maintain a Cassie state that exhibits excellent droplet mobility, yet their lower nucleation capability stems from a higher nucleation energy barrier.^18–23^ Ultimately, maximizing heat and mass transfer relies on an optimal interplay between these states, in which a surface must simultaneously promote active droplet nucleation and rapid droplet removal to ensure continuous surface renewal.

Recently, numerous studies have explored biphilic surfaces that integrate hydrophilic and hydrophobic regions to combine favorable nucleation with efficient droplet removal. Such surfaces are commonly engineered through spatial control of surface chemistry or patterned wettability.^24–28^ Liu et al.^29^ developed an armored superhydrophobic surface that enhanced condensate droplet removal and exhibited long-term resistance to condensation-induced wetting transitions. Wang et al.^30^ developed SiO_2_/TiO_2_-coated hybrid surfaces whose wettability could be regulated by UV exposure, demonstrating another route for controlling surface wettability.

Polydopamine (PDA) coatings can adhere to a variety of substrates, making the treated surfaces more hydrophilic through dopamine self-polymerization under alkaline conditions in a pure-oxygen environment.^31–33^ This unique characteristic has revealed new opportunities across various research fields, including membrane separation, biomedical technology, and composite materials.^34–37^ Microscale or larger hydrophilic patterns formed by PDA deposition have been investigated for water-harvesting applications. Zhang et al.^35^ reported a one-step, mask-free method for designing a superhydrophobic surface composed of uniform hydrophilic PDA arrays via inkjet printing, with a minimum feature size of 200 μm. Zhu et al.^38^ reduced the surface energy of the dopamine solution by depositing 5 μL droplets onto the surface, resulting in circular PDA patterns approximately 3.2 mm in diameter. However, these studies mainly relied on predefined hydrophilic patterns with controlled sizes, shapes, and spatial arrangements to capture and transport externally supplied fog droplets.

In contrast, temporal control of PDA deposition enables continuous tuning of surface wettability without intentionally creating distinct micro- or millimeter-scale hydrophilic domains. In this study, the wettability of a SiO_2_ nanoparticle-textured superhydrophobic surface was systematically regulated by varying the PDA reaction time. Advancing and receding contact angles were measured to assess changes in surface wettability and droplet mobility. At the same time, nucleation density, condensation behavior, water-harvesting performance, and heat-transfer performance were systematically evaluated under varying PDA modification conditions. In particular, the Cassie-to-Wenzel transition of sessile droplets and the DWC-to-FWC transition during condensation were observed and compared under varying PDA-modification conditions. Through this approach, the effects of progressively increasing surface hydrophilicity on droplet nucleation, mobility, and overall condensation performance were systematically investigated.

## Experimental Section

### Sample Preparation

The silicon substrates, cut into 2.0 cm × 1.5 cm, were immersed in piranha solution at 120 °C for 30 min to remove surface-adsorbed organic compounds. The piranha solution was prepared by mixing 95% w/w sulfuric acid and 30% w/w hydrogen peroxide at a 7:3 volume ratio. The cleaned silicon substrates were then washed with deionized (DI) water and stored in a sample bottle filled with DI water. DI water with a resistivity ≥ 18.2 MΩ cm was obtained by dual distillation followed by purification using PURELAB equipment (ELGA, LabWater).

The preparation of PDA0 (SHB) and the synthesis of the SiO_2_ nanoparticles were carried out as described in previous studies.^39,40^ The morphology of the synthesized SiO_2_ nanoparticles is shown in Figure S1. The diameter of SiO_2_ nanoparticles was 100.4 ± 14.4 nm, as determined by averaging over 305 nanoparticles in the SEM images (Figure S1). The sol–gel precursor was prepared using absolute ethanol (99.9%, Merck), DI water, tetraethylorthosilicate (TEOS, Merck Chemical Co.), and Tween 80 (Acros Organics), with masses of 1.325, 0.518, 1.5, and 0.6075 g, respectively. Then, 150 μL of 37% HCl was added to the sol–gel precursor solution, and the mixture was stirred at 240 rpm for 3 h. The resulting sol–gel solution was degassed for 30 min to remove dissolved gases. The sol–gel thin film was coated onto a cleaned silicon substrate by spin-coating the sol–gel solution (80 μL) at 4000 rpm for 10 s, followed by thermal curing at 150 °C for 1 h to remove the solvent. Then, SiO_2_ nanoparticles were coated onto the sol–gel-coated surface by spin-coating a suspension of SiO_2_ nanoparticles prepared in absolute ethanol. Finally, the SiO_2_ nanoparticle-textured surfaces were heated slowly at 1 °C/min from room temperature to 600 °C, then held at 600 °C for 30 min to remove residual solvent and stabilize the coating.

In preliminary experiments, a superhydrophobic surface was prepared by immersing the SiO_2_ nanoparticle-coated substrate in an 1 wt % solution of trichloro­(1H,1H,2H,2H-perfluorooctyl)­silane (FTS) in iso-octane for 2 h. The FTS-modified SiO_2_ nanoparticle-coated surfaces exhibited advancing contact angle (ACA), receding contact angle (RCA), and sliding angle (SA) of 157.8 ± 1.0°, 155.8 ± 1.6°, and 2.4 ± 0.4°, respectively. After further modification of the FTS-modified substrate with a 0.25 g/L dopamine solution for 25 min, the ACA decreased to 146.8 ± 2.2°, and the RCA fell below 30°, indicating pronounced contact-angle hysteresis and droplet pinning. However, after 6 h of vertical condensation process, the ACA and RCA recovered to 158.1 ± 0.8° and 154.3 ± 3.6°, respectively, indicating poor stability of the PDA coating on the FTS-modified surface. To improve PDA adhesion while retaining superhydrophobicity, substrates prepared using different OTS reaction times were subsequently examined. As shown in Figure S2, the ACA and RCA increased, while the SA decreased with increasing OTS reaction time, indicating a gradual increase in surface hydrophobicity. Based on these results, an OTS reaction time of 10 min was selected to prepare the superhydrophobic control surface, designated PDA0 (SHB).

For OTS modification, the SiO_2_ nanoparticle-coated substrates were immersed in piranha solution at 120 °C for 30 min, washed with DI water, and blown dry with nitrogen gas. Iso-octane (99%, Merck) was dehydrated by passing it through an anhydrous aluminum oxide column (Merck). Trichloro­(octadecyl)­silane (OTS, Sigma-Aldrich) was dissolved in iso-octane to form a 0.6 wt % OTS solution before use. All steps of the OTS silanization reaction were carried out in a nitrogen-filled glovebag to prevent hydrolysis caused by ambient moisture. The substrates were plasma-treated at 400 mTorr for 3 min and then immersed in a 0.6 wt % OTS solution for 10 min. Plasma treatment was applied to activate surface hydroxyl groups, thereby promoting the uniform formation of self-assembled monolayers. After silanization, the substrates were sequentially immersed in dichloromethane (99.5%, Merck), chloroform (99.0–99.4%, Merck), and absolute ethanol to terminate the reaction and remove unreacted species. The resulting superhydrophobic surface was designated PDA0 (SHB).

A 10 mM Tris-base buffer solution (pH 8.5) was prepared by dissolving Tris­(hydroxymethyl) aminomethane (Tris-base, J. T. Baker) in DI water. A dopamine solution at a concentration of 0.25 g/L was prepared by dissolving dopamine hydrochloride (Merck) in the 10 mM Tris-base buffer solution at room temperature. The samples were immersed in a stirred dopamine solution at 200 rpm in a pure-oxygen-filled glovebag for a prescribed reaction time. The samples were placed face down on an acrylic shelf in the dopamine solution to prevent large PDA aggregates from precipitating onto the surface.^41^ The fabrication process for the PDA-modified surfaces is illustrated in Figure 1. The PDAt notation was used to designate a PDA-modified surface, where “t” indicates the PDA reaction time in minutes. After PDA modification, the samples were washed with DI water and dried with N_2_.

**Figure 1:**
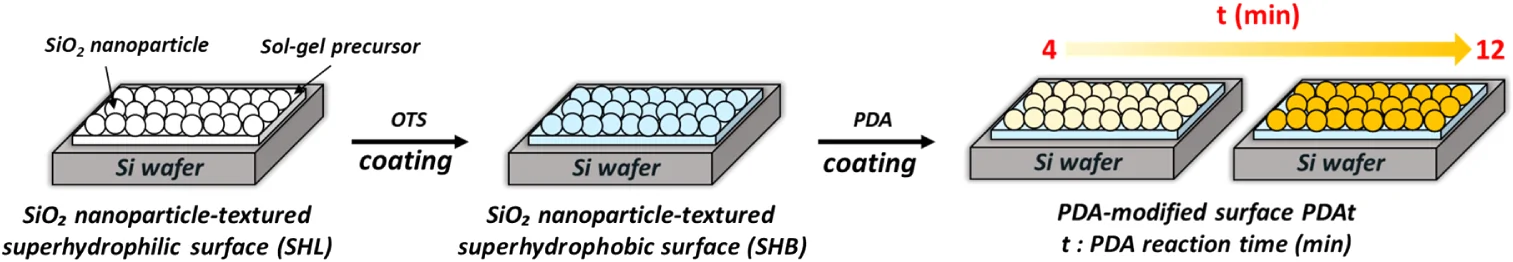
Schematic illustration of the fabrication of PDA-modified surfaces.

### Contact Angle Measurements

We applied an in-house-designed embedded-needle system, integrated with enhanced video microscopy and digital image analysis (MATLAB), to measure the advancing contact angles (ACA) and receding contact angles (RCA) of a water droplet on these substrates, as shown in Figure S3. A detailed description of the experimental procedure is available in our previous work.^2,42,43^ A water droplet of 3 μL was injected using a syringe pump (model NE-1000, NEW ERA) and deposited on the surface. The total water volume reached 8 μL at a constant injection rate of 0.8 μL/min during the ACA measurement. The RCA measurement was conducted by withdrawing water from the liquid droplet at a removal rate of 0.8 μL/min. The ACA and RCA were obtained via digital image processing, which determines the angle between the fitted outline of the water droplet’s bottom and the baseline from side-view images recorded by a CCD camera. The CCD camera’s frame rate was set to 10 frames per second. The ACA/RCA measurements were performed at least three times in different regions for each sample to determine the average and standard deviation, as reported in the data.

The sliding angle measurement setup consisted of a motorized rotation stage (08MRA-3, Unice E-O Service Inc.) and computer-controlled software. An 8 μL water droplet was initially placed on a horizontal surface, and its ability to roll off the surface was observed at a slow angular speed of 1°/s. The sliding angle (SA) was recorded when the water droplet began to move. A pinned droplet was defined as one that failed to detach from the surface even at a 90° tilt. In addition, a bottom-view camera (GS3-U3–23S6M-C, FLIR) integrated with an inverted optical microscope was used to directly observe the wetting state of droplets placed on the surface and to verify the wetting behavior of condensed droplets after 2 h of condensation. Detailed experimental procedures are available in our previous work.^4^

### Condensation Experiments

A condensation experiment was conducted in both horizontal and vertical orientations, depending on the cooling plate’s tilt angle (CAP500, Linkam). Figures S4 and S5 schematically illustrate the experimental setup for the horizontal and vertical condensation, respectively. Droplet dynamics during horizontal condensation were observed using optical microscopy (BXFM, Olympus) at ×20 magnification (562.56 μm × 351.6 μm) and captured by a CCD camera at 10 frames/s. The surface nucleation density was analyzed using two independently prepared samples, with one measurement per sample. The ambient temperature (T) and relative humidity (RH) were controlled at 21.4 ± 1.3 °C and 53.6 ± 3.8%, respectively. In addition, vertical condensation was conducted to evaluate water-collection efficiency and droplet mobility in a high-relative-humidity environment (T = 29.9 ± 0.6 °C and RH = 96.5 ± 0.6%). A CCD camera also recorded the condensation process at 3 frame/s. During condensation, liquid nitrogen and a temperature controller (T96, Linkam) were used to cool the system plate at a rate of −20 °C/min. The cooling plate was then maintained at a constant temperature of 0 °C, with the surface temperature (on top of the patterned substrates) of 2.0 ± 0.2 °C. The amount of condensed water was collected at 1, 2, and 3 h into the vertical condensation process. Three repeated water-collection measurements were performed using two independently prepared samples: one sample was measured twice, and the other once. The recorded video was used for further image analysis in ImageJ.

## Results and Discussion

### Surface Characterization of PDA-Modified Surfaces

To confirm the deposition and distribution of PDA on the PDA-modified surfaces, SEM, XPS, AFM, and elemental mapping analyses were performed. Figure S6 shows SEM images of the surfaces of PDA0 (SHB), PDA12, and PDA60. A thin layer of PDA was observed covering the SiO2 nanoparticles on PDA60 (prepared with a long PDA reaction time of 60 min), as shown in Figure S6­(c), resulting in a relatively thick PDA coating on the SiO_2_ nanoparticles and a less distinct nanoparticle surface morphology. However, at shorter PDA reaction times, PDA deposition could not be clearly identified from the SEM images. As shown in Figure S6­(b), the surface morphology of PDA12 was difficult to distinguish from that of PDA0 (SHB).

The AFM phase images exhibited a uniform phase distribution, indicating a uniform PDA distribution across the modified surface, as shown in Figure S7­(a). Meanwhile, the elemental mapping results in Figure S7­(b) showed that the element N, a characteristic element associated with PDA, was uniformly distributed throughout the observation area. The AFM and elemental mapping results demonstrated that PDA was uniformly distributed over the PDA-modified surfaces.

As shown in Figure 2(a), the XPS survey spectra revealed that the N 1s peak gradually increased with increasing PDA reaction time, whereas the intensities of the Si 2s and Si 2p peaks decreased. These results indicated that PDA progressively covered the original OTS-coated SiO_2_ nanoparticle-textured surface as the PDA reaction time increased. Furthermore, the N 1s high-resolution XPS spectra in Figure 2(b) showed, as expected, no obvious N 1s peak for PDA0 (SHB). In contrast, no distinct characteristic N 1s peak could be resolved for PDA4 due to the relatively low amount of deposited PDA. In contrast, PDA10 and PDA60 exhibited characteristic signals at approximately 401.2 and 399.4 eV, corresponding to −NH_2_ and −NH, respectively.^44,45^ The corresponding C 1s and O 1s high-resolution XPS spectra are shown in Figure S8­(a) and S8­(b), respectively. In the C 1s XPS spectra, peaks at approximately 286.4 and 284.8 eV were assigned to C–O/C–N and C–C/C–H, respectively.^44,45^ In the O 1s XPS spectra, peaks at approximately 532.5 and 530.7 eV were attributed to Si–O–Si and C=O, respectively.^44,46^ These PDA-related characteristic signals became more pronounced as the PDA reaction time increased, further confirming the successful deposition of PDA on the surface.

**Figure 2:**
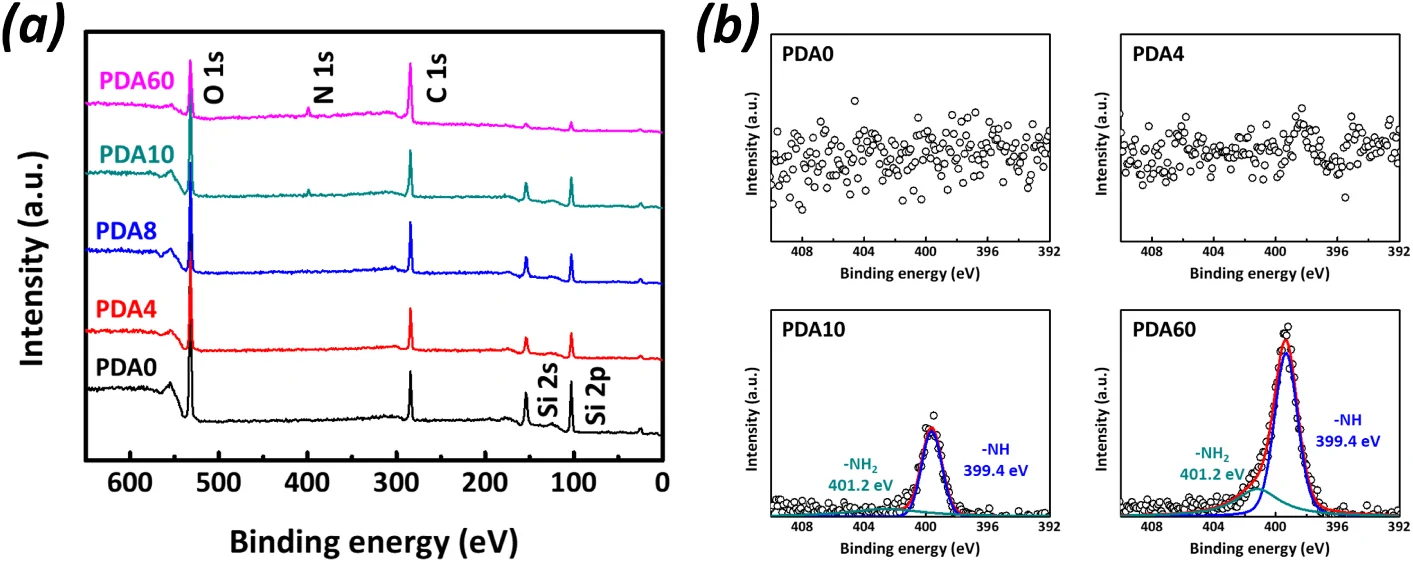
(a) XPS survey spectra of PDA-modified surfaces: PDA0 (SHB), PDA4, PDA8, PDA10, and PDA60; and (b) N 1s high-resolution XPS spectra of PDA-modified surfaces: PDA0 (SHB), PDA4, PDA10, and PDA60.

### Contact Angle Measurements and Wetting States of a Sessile Droplet on PDA-Modified Surfaces

The PDA-modified surfaces were fabricated from the PDA0 (SHB) surface and then subjected to a PDA-induced hydrophilic treatment. The ACA, RCA, and SA of the PDA0 (SHB) were 154.1 ± 1.0°, 151.3 ± 1.2°, and 7.7 ± 3.5°, respectively, as shown in Figure S2. Figure 3 and Table S1 present the contact angle analysis of PDA0 (SHB) and PDA-modified surfaces at PDA reaction times of 0, 4, 8, 10, and 12 min. The wetting behavior of sessile droplets on the PDA0 (SHB), PDA4, and PDA8 substrates (i.e., the PDA reaction time <9 min) consistently exhibited the Cassie state. Although the ACA and RCA decreased gradually as the PDA reaction time increased, the PDA4 and PDA8 surfaces still maintained superhydrophobic properties with low sliding angles. In contrast, the wetting behavior of a sessile water droplet on the PDA10 and PDA12 substrates (i.e., PDA reaction time >9 min) exhibited the Wenzel state. The RCA of water droplets on the PDA10 and PDA12 substrates was <20°, indicating sufficient PDA deposition to induce strong droplet pinning. The low contact angle hysteresis of the Cassie droplets indicated high droplet mobility, whereas the high contact angle hysteresis of the Wenzel droplets indicated strong droplet pinning. The transition boundary of the wetting state from Cassie (observed on the PDA8 substrate) to Wenzel (observed on the PDA10 substrate) was estimated at a PDA reaction time of approximately 9 min, based on the two adjacent PDA reaction times of 8 and 10 min.

**Figure 3:**
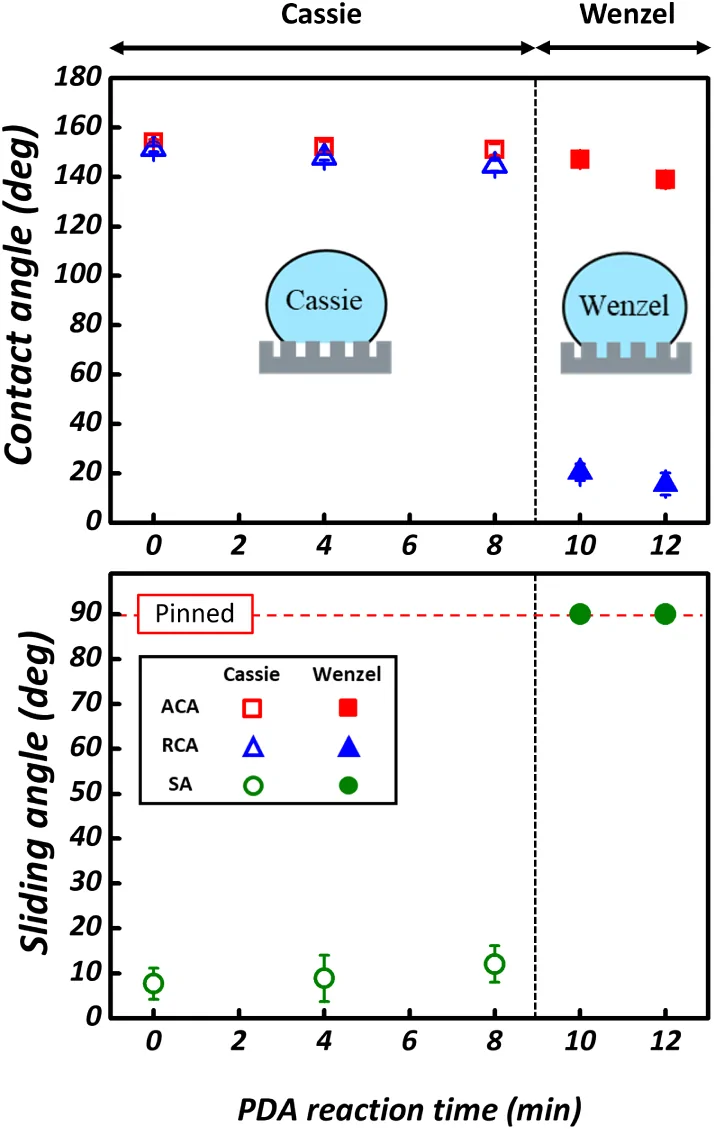
The ACA, RCA, and SA of sessile droplets on OTS-modified surfaces at different PDA reaction times. The red square, blue triangle, and green circle symbols represent the ACA, RCA, and SA, respectively. The open and filled symbols represent the Cassie and Wenzel droplets, respectively.

The SiO_2_ nanoparticle-textured surface without OTS coating (SHL) exhibited superhydrophilic wetting behavior. When a droplet contacted the SHL surface, it rapidly spread and infiltrated the SiO_2_ nanoparticle-textured surface, forming an almost zero-contact-angle, fully wetted state. The droplets transformed into a continuous liquid film within a short time, rather than remaining discrete.

In comparison, the FTS-modified superhydrophobic surface required a longer PDA reaction time to induce the Cassie-to-Wenzel transition, which occurred at approximately 22.5 min (Figure S9). This result indicated that PDA modification alters the wettability of the OTS-modified surface more effectively than that of the FTS-modified surface.

### Horizontal Condensation Process: Growth Mechanism and Wetting Behavior of Condensed Droplets on PDA-Modified Surfaces

In contrast to the wetting behavior of a sessile droplet on patterned surfaces, a condensed droplet may exhibit distinct wetting characteristics due to the dynamic condensation process. The detailed mechanism and wetting behavior of condensed droplets on the hydrophobic square-pillar-array-patterned surfaces have been extensively discussed in our previous studies.^2,4^ In this study, we aim to investigate the effect of PDA coating on the growth mechanism and wetting behavior of condensed droplets on PDA-modified surfaces. Water vapor nucleated at random sites on the surface grows independently and eventually coalesces with adjacent water droplets to form larger droplets, as shown in the optical microscopy images of condensed droplets (nuclei) on the superhydrophobic surface (PDA0 (SHB)) and two PDA-modified surfaces (PDA8 and PDA12) captured at the early stage of the condensation process, as shown in Figures 4 and S10.

**Figure 4:**
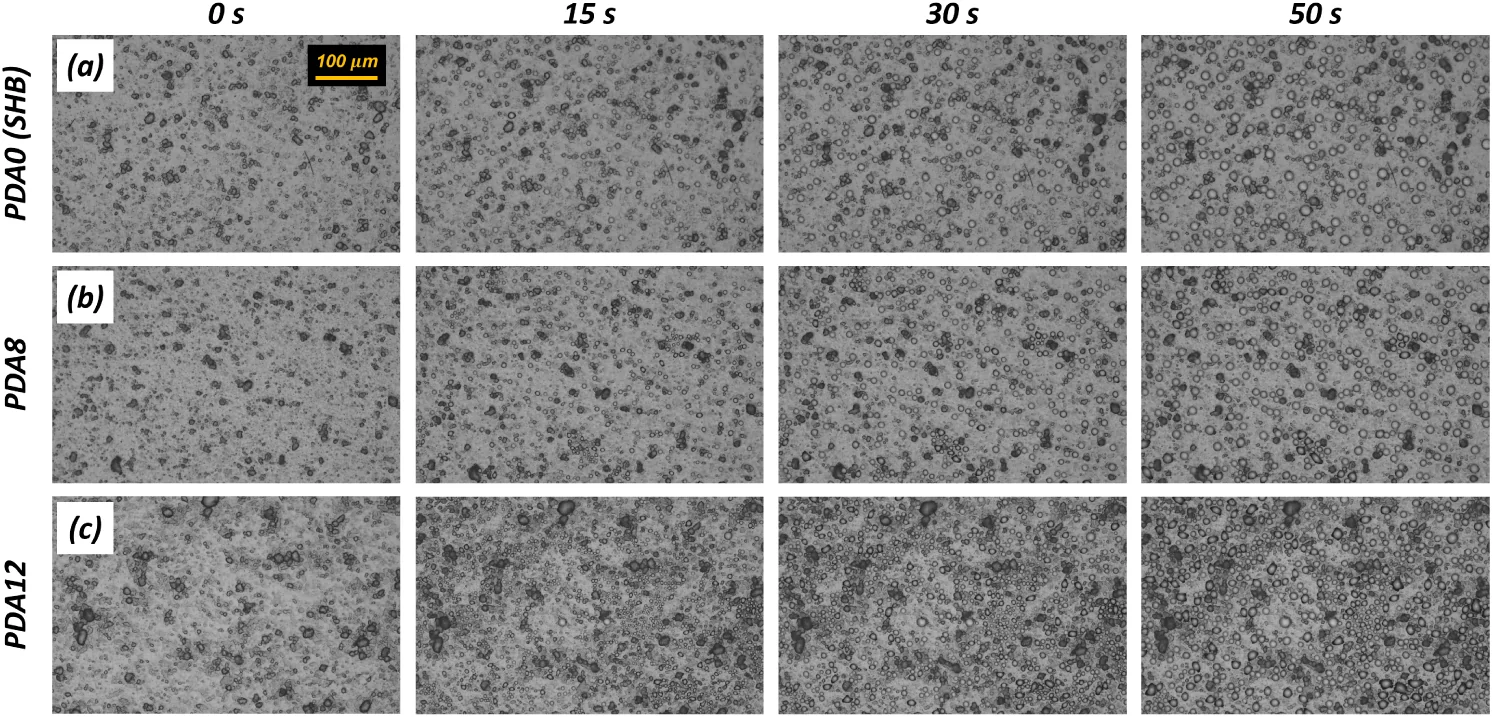
Illustration of the OM images during horizontal condensation on surfaces: (a) PDA0 (SHB), (b) PDA8, and (c) PDA12 taken at 0, 15, 30, and 50 s.

The statistical results of the number density, surface coverage, and droplet diameter of condensed droplets on surfaces are shown in Figure 5. Droplet number density provided insight into how PDA modification influenced condensate formation during nucleation, growth, and coalescence. Figure 5(a) shows that the droplet number density markedly increased with increasing PDA reaction time. During droplet growth, droplets coalesced upon collision with neighboring droplets, leading to a gradual decrease in droplet number density. This phenomenon was more pronounced on surfaces with initially high droplet number density, such as PDA10 and PDA12.

**Figure 5:**
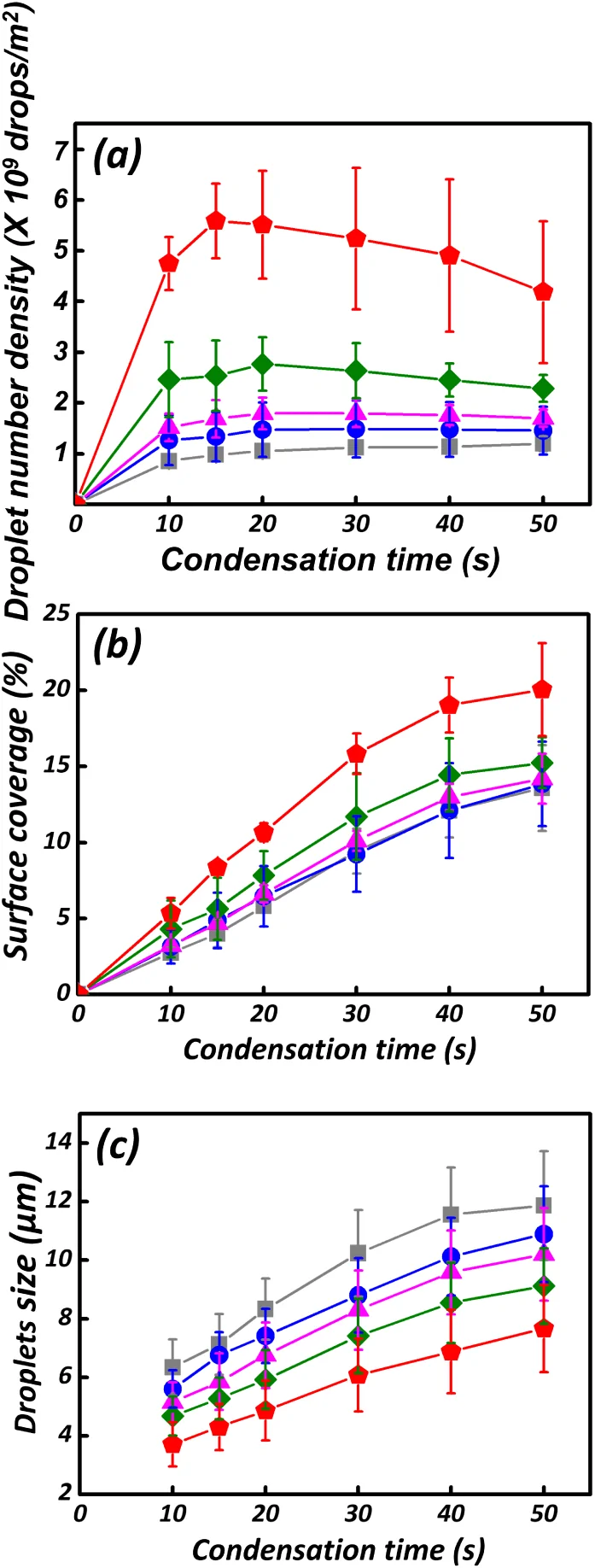
Horizontal condensation profiles on surfaces: (a) droplet number density, (b) surface coverage, and (c) droplet size. The gray square, blue circle, pink triangle, green diamond, and red pentagon symbols represent PDA0­(SHB), PDA4, PDA8, PDA10, and PDA12, respectively.

In contrast, the higher nucleation energy barrier on PDA0 (SHB), PDA4, and PDA8 substrates limited water vapor nucleation, thereby reducing the likelihood of droplet collisions and coalescence during growth. As a result, the droplet number density gradually increased and eventually reached a relatively stable value within the 50 s observation period. However, PDA deposition reduced the local nucleation energy barrier on the modified surfaces, thereby increasing the probability of water vapor condensation and enhancing the number density of condensed droplets.

Surface coverage analysis revealed that droplet-occupied areas on all PDA-modified surfaces exceeded those on PDA0 (SHB), as shown in Figure 5(b). These findings suggest that increased surface hydrophilicity enhances nucleation and promotes water vapor condensation. Figure 5(c) presents the average droplet diameter. As surface hydrophilicity increased, the size of condensed droplets decreased, while droplet number density increased significantly. This phenomenon is attributed to the increased nucleation density on more hydrophilic surfaces.

Figure 6 shows the variation in the size distribution of condensed droplets during the initial condensation process on surfaces prepared at different PDA reaction times. For statistical analysis, we divided the condensed droplets into three size groups: *d* < 5 μm, 5 < *d* < 10 μm, and 10 < *d* < 20 μm (where d is the diameter of the condensed droplet). On the PDA0 (SHB) surface, the condensed droplets observed in the first 50 s were mainly concentrated in the size ranges of 5 < *d* < 10 μm and 10 < *d* < 20 μm. As PDA reaction time increased, the number of droplets with *d* < 5 μm increased significantly during the first 15 s, indicating that enhanced PDA modification promoted droplet nucleation. For the PDA12 surface, the size distribution of condensed droplets shifted significantly toward smaller sizes, with a significant increase in the number of droplets with *d* < 5 μm. This shows that the higher degree of PDA modification on the PDA12 surface significantly reduced the nucleation energy barrier, promoting the formation of more small droplets. Overall, PDA modification effectively modulates surface wettability, thereby affecting the size distribution and droplet number density of condensed droplets. The effect of surface wettability on nucleation behavior has been investigated in several studies.^8,18,19,30^

**Figure 6:**
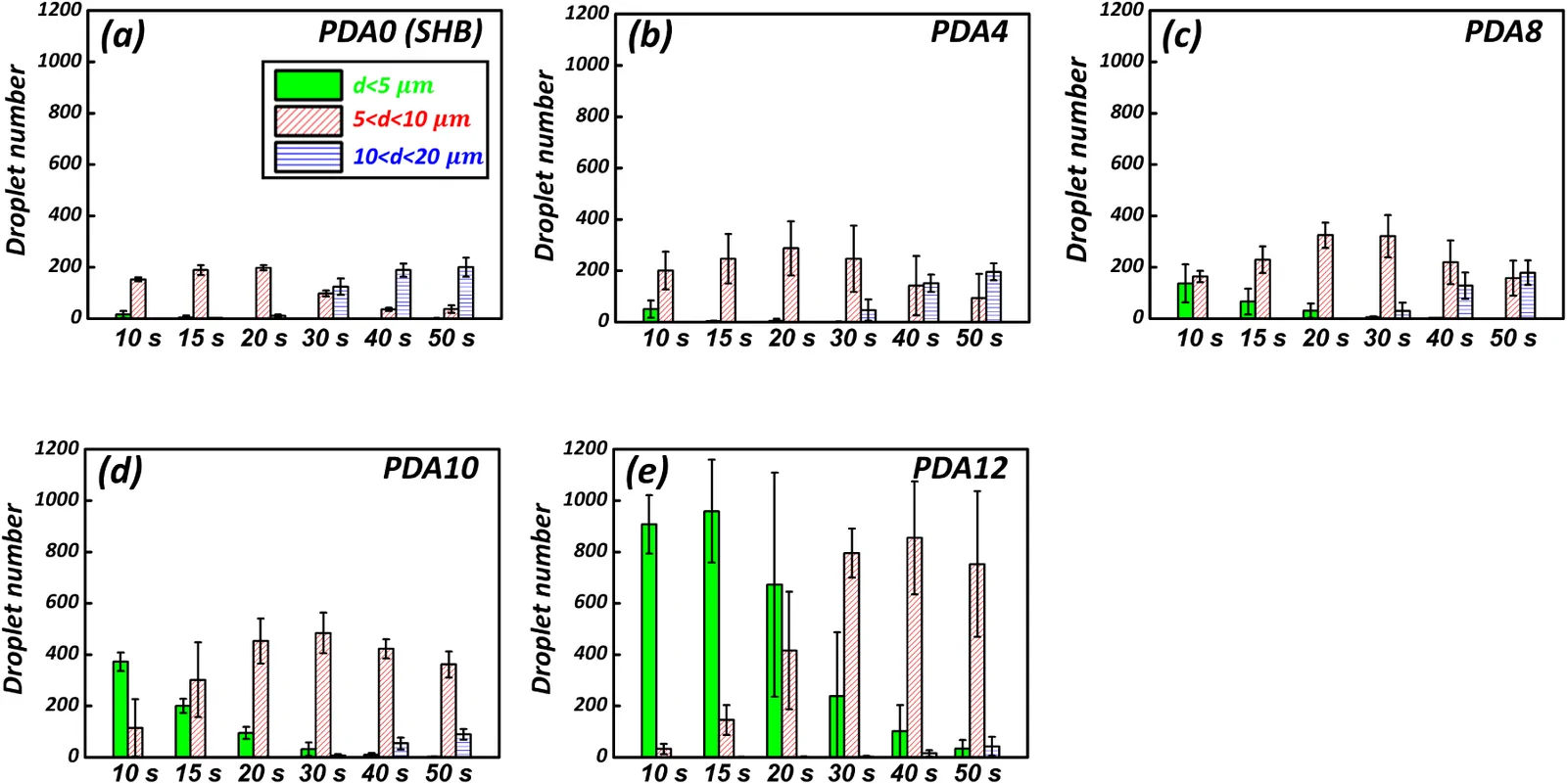
The variation of condensed droplet size distribution on surfaces: (a) PDA0 (SHB), (b) PDA4, (c) PDA8, (d) PDA10, and (e) PDA12 as a function of time (at 10, 15, 20, 30, 40, and 50 s).

Bottom-view images were used to further verify the wetting states of sessile droplets on the surfaces. On the PDA0 (SHB) and PDA4 surfaces, droplets exhibited the Cassie state, characterized by a relatively small actual contact area. The images displayed a spotted pattern of bright and dark regions, with dark regions corresponding to the actual liquid–solid contact areas and bright regions indicating the air–liquid interface, as shown in Figure 7(a) and 7(b). In contrast, an 8 μL water droplet was pinned during the sliding-angle measurement on PDA10 and on the OTS-modified surface prepared with a reaction time of 30 s (OTS0.5). The ACA and RCA of OTS0.5 were 148.9 ± 2.6° and 34.9 ± 3.3°, respectively, as shown in Figure S2. Water droplets on both surfaces exhibited the Wenzel state, characterized by a significantly larger contact area, which appeared as a uniformly dark region in the bottom-view image, as shown in Figure 7(c) and 7(d). In Figure 7, the red dashed lines represent the maximum droplet diameter, and the yellow dashed lines indicate the contact diameter at the liquid–solid interface.

**Figure 7:**
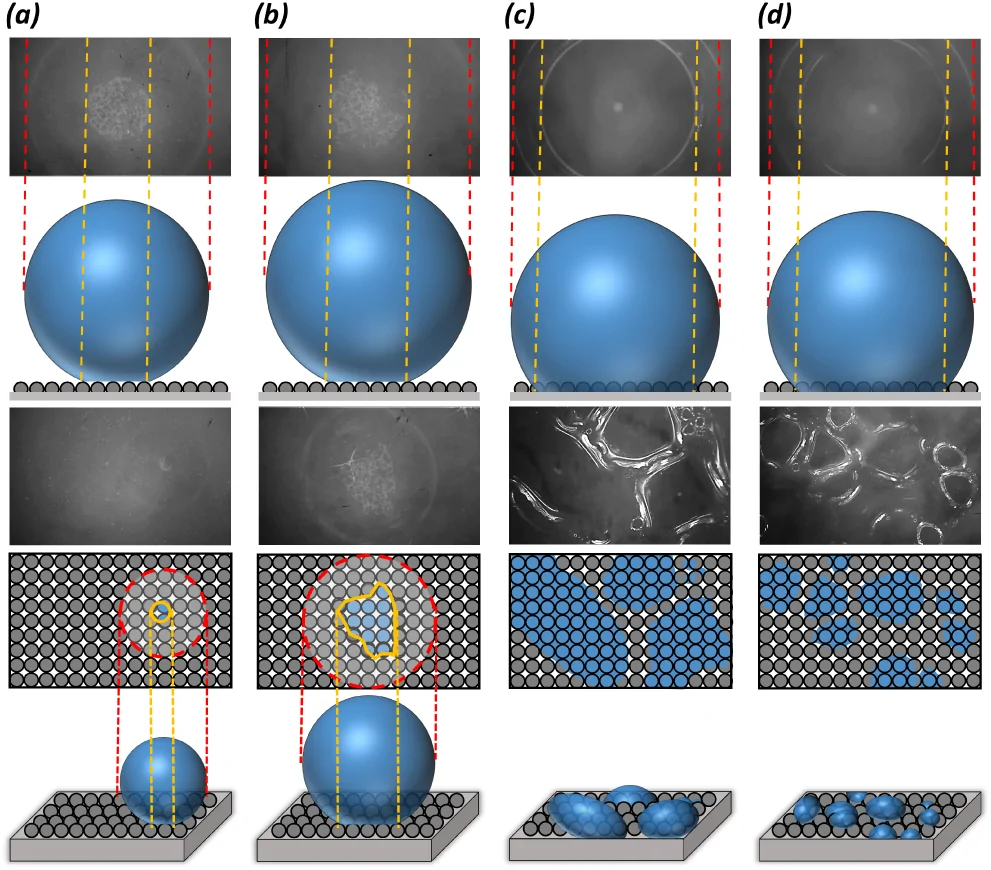
The bottom-view (the top row) and side-view schematic illustration (the second row) of a sessile droplet deposited on the surfaces, bottom-view image (the third row), bottom-view schematic illustration (the fourth row), and tilted-view schematic illustration (the bottom row) of condensed water droplets after 2 h of condensation process on the surfaces: (a) PDA0­(SHB), (b) PDA4, (c) PDA10, and (d) OTS0.5.

After 2 h of horizontal condensation experiments, the wetting behavior of the condensed droplets on the surface was carefully examined using a bottom-view camera. The condensed droplets still exhibited a Cassie state on the PDA0 (SHB) and PDA4 surfaces, as shown in Figure 7(a) and 7(b). On the other hand, the condensed droplets on the PDA10 and OTS0.5 surfaces exhibited an irregular Wenzel state with an irregular shape of three-phase-contact-line (i.e., a significant deviation from a circular shape), as illustrated in Figure 7(c) and 7(d).^2,4^

### Vertical Condensation Process

In comparison with horizontal condensation processes, vertical condensation processes involve more complex droplet dynamics, including nucleation, growth, coalescence, and droplet departure. Moreover, the condensation mode (DWC and FWC) is a key factor affecting water-harvesting and heat-transfer efficiency.

Figure 8 shows optical images of the morphology of condensed droplets on six substrates (PDA0 (SHB), PDA4, PDA8, PDA10, PDA12, and SHL) at 5, 15, 30, 45, and 60 min during a 1-h condensation process. The DWC was consistently observed on the PDA0 (SHB), PDA4, PDA8, and PDA10 substrates. Videos S1, S2, S3, and S4 show the 1 h condensation process on the PDA0 (SHB), PDA4, PDA8, and PDA10 substrates, respectively. All condensed droplets on the PDA12 or SHL substrate coalesced into a continuous water film that covered the entire surface within 30 min (see Figure 8(e) and 8(f) and Videos S5 and S6). Therefore, a transition of condensation mode from DWC (observed on the PDA10 substrate) to FWC (observed on the PDA12 substrate) was estimated to occur on the substrate prepared at a PDA reaction time of approximately 11 min based on condensation modes observed on the substrates prepared at these two adjacent PDA reaction times, 10 and 12 min.

**Figure 8:**
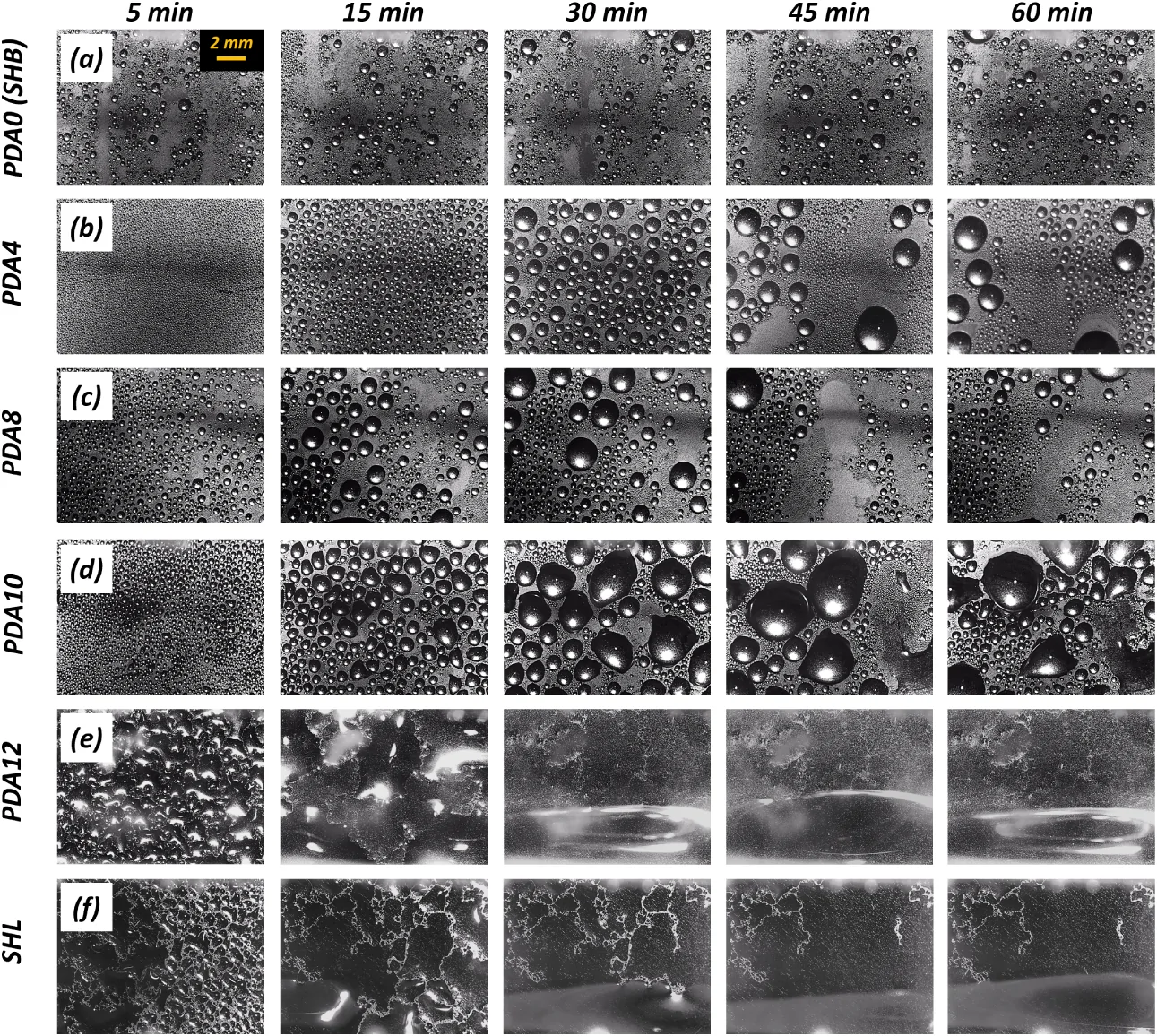
Evolution of optical images of the vertical condensation process on surfaces: (a) PDA0­(SHB), (b) PDA4, (c) PDA8, (d) PDA10, (e) PDA12, and (f) SHL. Optical images of size 14.08 mm × 10.58 mm were taken during the condensation process at 5, 15, 30, 45, and 60 min.

The condensed Cassie droplets on the PDA0 (SHB), PDA4, and PDA8 substrates adopt a spherical shape due to reduced surface free energy and minimized liquid surface area, as illustrated in the optical images in Figure 8(a)-8(c). Note that a superhydrophobic surface is not a necessary condition for a system to exhibit the DWC in the vertical condensation experiments. Interestingly, as shown in Figure 8(d), the wetting behavior of condensed droplets on the PDA10 substrate always exhibits a Wenzel state, forming an irregularly shaped three-phase-contact-line (strongly deviating from a circular shape) due to strong pinning. Notably, the condensed Wenzel droplets maintain “limited” mobility and exhibit DWC rather than FWC on the PDA10 substrate; this phenomenon is termed irregular Wenzel DWC. Figure 9 illustrates the growth and merging of two adjacent Wenzel droplets, which ultimately leads to droplet sliding (see Video S4 for details). This droplet motion is called coalescence-induced sliding. On the PDA10 substrate, Wenzel droplet mobility is governed primarily by coalescence-induced sliding, thereby enabling DWC.

**Figure 9:**
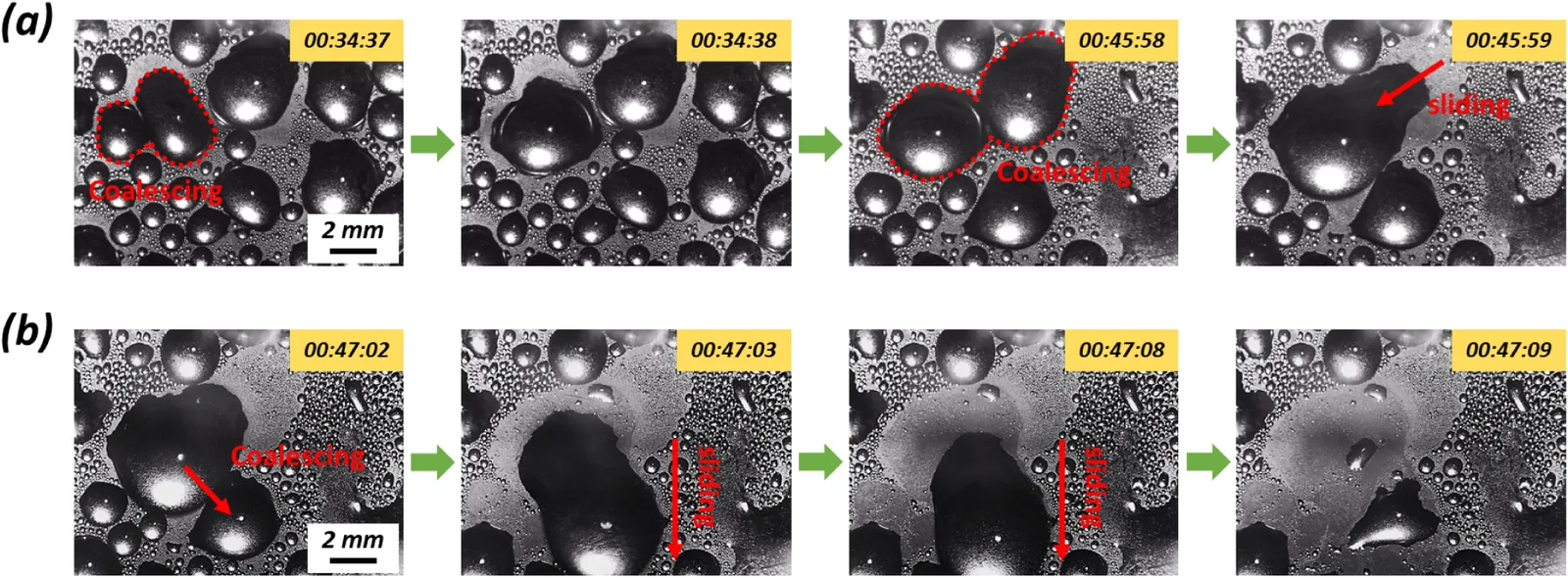
(a) and (b) Evolution of droplet mobility via coalescence-induced sliding on the PDA10 substrate (these images were extracted from Video S4).

It was reported that both partially wetted and suspended droplets on superhydrophobic nanostructured surfaces can undergo coalescence-induced jumping.^47,48^ This behavior occurs because the low surface free energy of the superhydrophobic surface minimizes liquid–solid interactions, allowing the surface energy released during coalescence to be efficiently converted into the kinetic energy required for jumping. Compared with Cassie-state droplet coalescence, Wenzel droplet coalescence involves greater energy dissipation due to stronger liquid–solid adhesion. Consequently, due to increased energy dissipation and the high Gibbs free-energy barriers of Wenzel droplets, a higher coalescence energy conversion is required to induce droplet jumping.^49^ This jumping motion removes small condensed droplets from the surface at an early stage, enabling new nuclei to continuously form and grow, thereby greatly reducing the average droplet size and promoting continuous surface renewal.

In this study, jumping behavior was also observed on the PDA0 (SHB) substrate. We observed two types of jumping behavior. The first type was multidroplet-coalescence-induced jumping, in which the merged droplet detaches from the surface, as shown in Figure 10(a) and Video S7. The second type involved multidroplet coalescence, in which droplets jump over neighboring droplets and subsequently collide with droplets farther away, as shown in Figure 10(b) and Video S8. Figure 10(c) shows the droplet departure induced by multidroplet coalescence. As multiple droplets collide and coalesce into a larger one, the system’s total surface energy decreases, releasing free energy that is subsequently converted into kinetic energy, thereby driving the droplet to slide off the surface. Figure 10(d) illustrates a droplet that grew and coalesced with neighboring droplets until gravity overcame the liquid–solid adhesion, causing it to depart from the surface.

**Figure 10:**
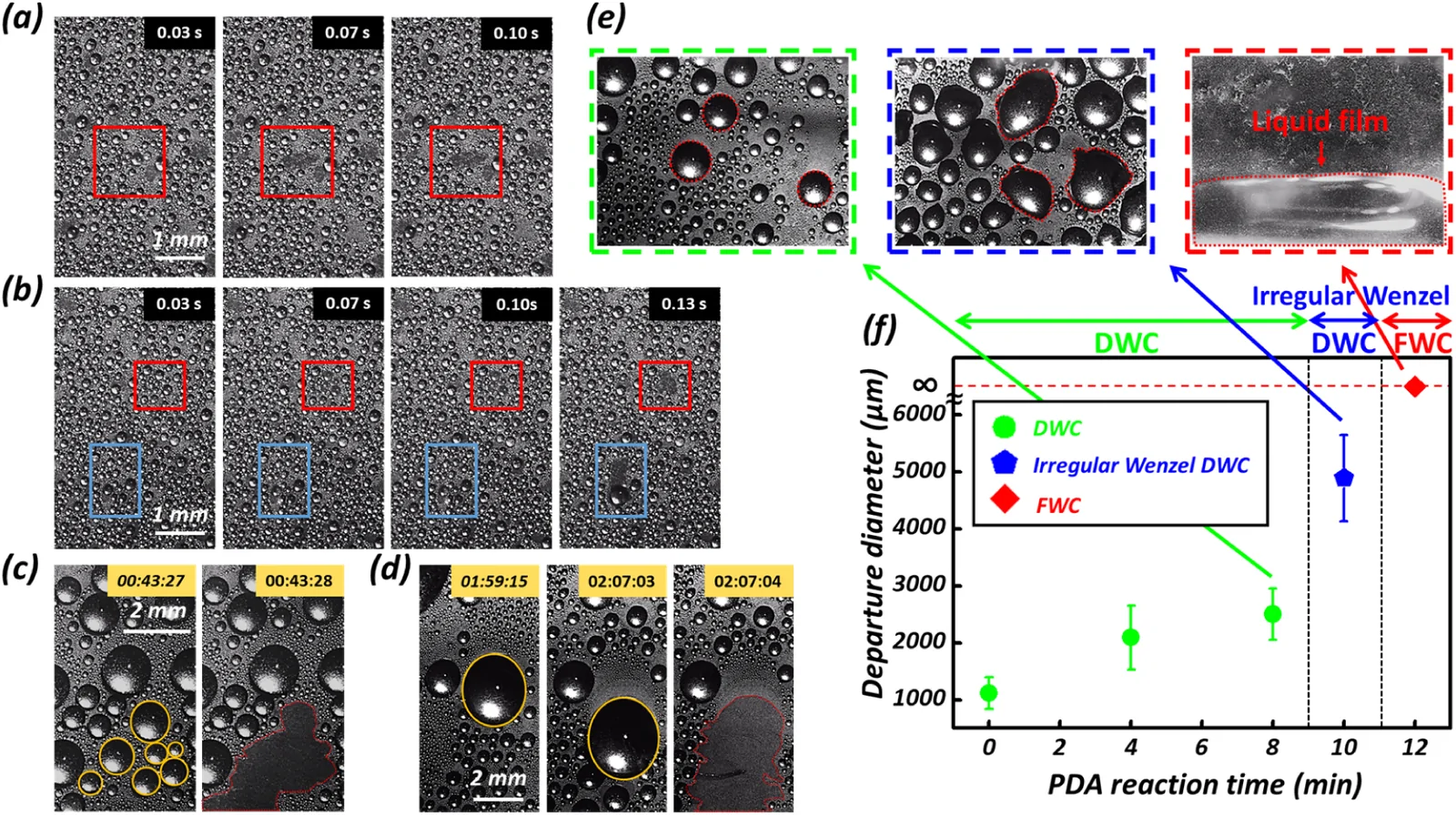
Optical images of multidroplet-coalescence-induced (a) the detachment of the droplets by jumping and (b) droplets jumping over neighboring droplets and colliding with others on the PDA0 (SHB) substrate; (c) multidroplet-coalescence-induced departure event observed on the PDA4 substrate, (d) natural departure behavior observed on the PDA8 substrate, and (e) and (f) natural departure diameters observed on the surfaces with different PDA reaction times. Note that the condensation mode for the substrates prepared in the 0 min/9 min window of PDA reaction time exhibits Cassie DWC. On the other hand, the condensation mode for the substrates prepared in the 9 min/11 min/PDA10 window of PDA reaction time exhibits irregular Wenzel DWC.

To evaluate the effect of PDA modification on droplet departure behavior, the departure diameter was defined as the droplet diameter immediately before natural departure from the surface. As PDA reaction time increased, the surface became more hydrophilic, reducing droplet mobility and gradually increasing the departure diameter. The departure diameter of the PDA0 (SHB) substrate was 1120 ± 280 μm, while those of the PDA4, PDA8, and PDA10 substrates were 2094 ± 561 μm, 2503 ± 452 μm, and 4895 ± 760 μm, respectively, as illustrated in Figure 10(f).

For small water droplets, gravity is negligible, and the shape of water droplets is then simply a spherical cap, achieving a small Bond number $B_{o}=\frac{\mathrm{\rho g}r^{2}}{\gamma }={\left(\frac{r}{\lambda_{c}}\right)}^{2}$ , which accounts for the ratio of gravitational and surface tension forces. Here ρ is the fluid density (= 995.84 kg/m^3^ at *T*_saturated_ = 29.3 °C), g is the gravitational acceleration (= 9.81 m/s^2^), r is the radius of the droplet, γ is the surface tension of water (= 71.31 mN/m at *T*_saturated_ = 29.3 °C), and *λ_c_* is the capillary length ( $=\sqrt{\gamma /\rho g}=2.701\mathrm{mm}$ at 29.3 °C). Then the departure diameters for the PDA0, PDA4, and PDA8 substrates were used as the droplet radius r to calculate their Bond numbers: 0.0456 ± 0.0224, 0.160 ± 0.083, and 0.221 ± 0.080, respectively. All these Bond numbers are rather small, indicating that surface tension dominates. All the condensed Cassie droplets on the PDA0, PDA4, and PDA8 substrates exhibit a spherical cap, as illustrated in Figure 8(a)-8(c) (also see Videos S1, S1, and S3).

On the other hand, the Bond number for the PDA10 substrate was 0.837 ± 0.258, with a rather large standard deviation. Note that the Bond number ranges from 0.50 to 1.26 for the PDA10 substrate, which is much closer to, and even larger than, unity; the gravity effect cannot be neglected. All large Wenzel droplets on the PDA10 substrate exhibit an irregular shape of three-phase-contact-line, as illustrated in Figure 10(e). The mobility of Wenzel droplets on the PDA10 substrate is primarily driven by coalescence-induced sliding and gravity. Therefore, the 9 min/11 min/PDA10 window of PDA reaction times is classified as an irregular Wenzel DWC in Figure 10(f).

In comparison, Zhu et al.^38^ reported superhydrophobic substrates with a hydrophilic pattern approximately 3.2 mm in diameter. The maximum droplet diameter observed on the hydrophobic area was 2.2 ± 0.4 mm, while that on the hydrophilic PDA areas was 6.4 ± 1.5 mm. This significant difference was attributed to the relatively large hydrophilic regions, resulting in a notable disparity in droplet departure diameters between hydrophilic and hydrophobic regions. In this study, we prepared PDA-modified surfaces with uniformly distributed PDA to determine a characteristic droplet departure diameter for each PDA-modified surface.

For the PDA12 substrate, the formation of a water film during condensation prevented the determination of its departure diameter. Thus, an infinite departure diameter was assigned to the PDA12 substrate, as shown in Figure 10(f). The observed large natural departure diameter on the PDA10 was attributed to the difference in droplet size between the sliding-angle measurement and the bottom-up condensation process, as illustrated in Figure S11­(a). The natural departure event was primarily gravity-driven and occurred when the instantaneous droplet diameter reached 4890 μm, corresponding to a droplet volume of 60.1 μL. The instantaneous droplet volume on the PDA10 substrate exceeded the droplet volume (8 μL) used in the sliding angle measurement. We also measured the sliding angle of droplets with different volumes on the PDA10 substrate, as shown in Figure S11­(b). When the droplet volume was small, the gravitational force was insufficient to overcome the adhesive force of the PDA coating, leaving the droplet pinned to the surface. When the droplet volume was ≥50 μL, the gravitational force could overcome the adhesive force, allowing the droplet to slide on the surface. Furthermore, as the droplet volume increased, the greater gravitational force enhanced sliding, leading to a gradual decrease in the measured sliding angle, as shown in Figure S11­(c).

Since the initial stage of condensation involves droplet nucleation and growth, droplets departed under gravity only after coalescing into larger ones. Therefore, condensation experiments were conducted for durations of 1, 2, and 3 h. After 6 h of condensation, the ACA and RCA showed no significant change compared to their precondensation values, as shown in Figure S12. To further evaluate the long-term stability and mechanical durability of the PDA-modified surfaces, the PDA10 surfaces were subjected to two tests separately: (1) continuous vertical condensation for 24 h; and (2) ultrasonication in 10 mL of DI water for 1 h. The corresponding high-resolution XPS spectra of C 1s, O 1s, and N 1s after these two tests are shown in Figures S13­(a) and S13­(b), respectively. The characteristic signals associated with PDA remained intact after both long-term condensation and ultrasonication treatments, indicating that the PDA coating was retained on the surface. These results indicate that the PDA exhibited good adhesion stability on the OTS-silanized superhydrophobic surface after 10 min of treatment. The total condensate mass collected per unit area (mg cm^–2^) was plotted against condensation time (h), and a linear regression was performed. The slope of the regression line represents the condensate mass collection rate, as shown in Figure 11(a). Figure 11(b) presents the condensate mass collection rates for PDA0 (SHB), PDA-modified surfaces, and SHL, defined as the total condensate mass collected per unit time and unit surface area (mg cm^–2^ hr^–1^). For the PDA0 (SHB), PDA4, PDA8, and PDA10 substrates, DWC was observed. As the PDA reaction time increased, the condensate mass collection rate increased. The condensate mass collection rates of the PDA0 (SHB), PDA4, PDA8, and PDA10 substrates were 211.2 ± 20.6, 227.1 ± 20.0, 283.8 ± 30.8, and 342.2 ± 33.7 mg cm^–2^ hr^–1^, respectively. Among the tested samples, PDA10 demonstrated the highest condensate mass collection rate, 62.0% higher than PDA0 (SHB).

**Figure 11:**
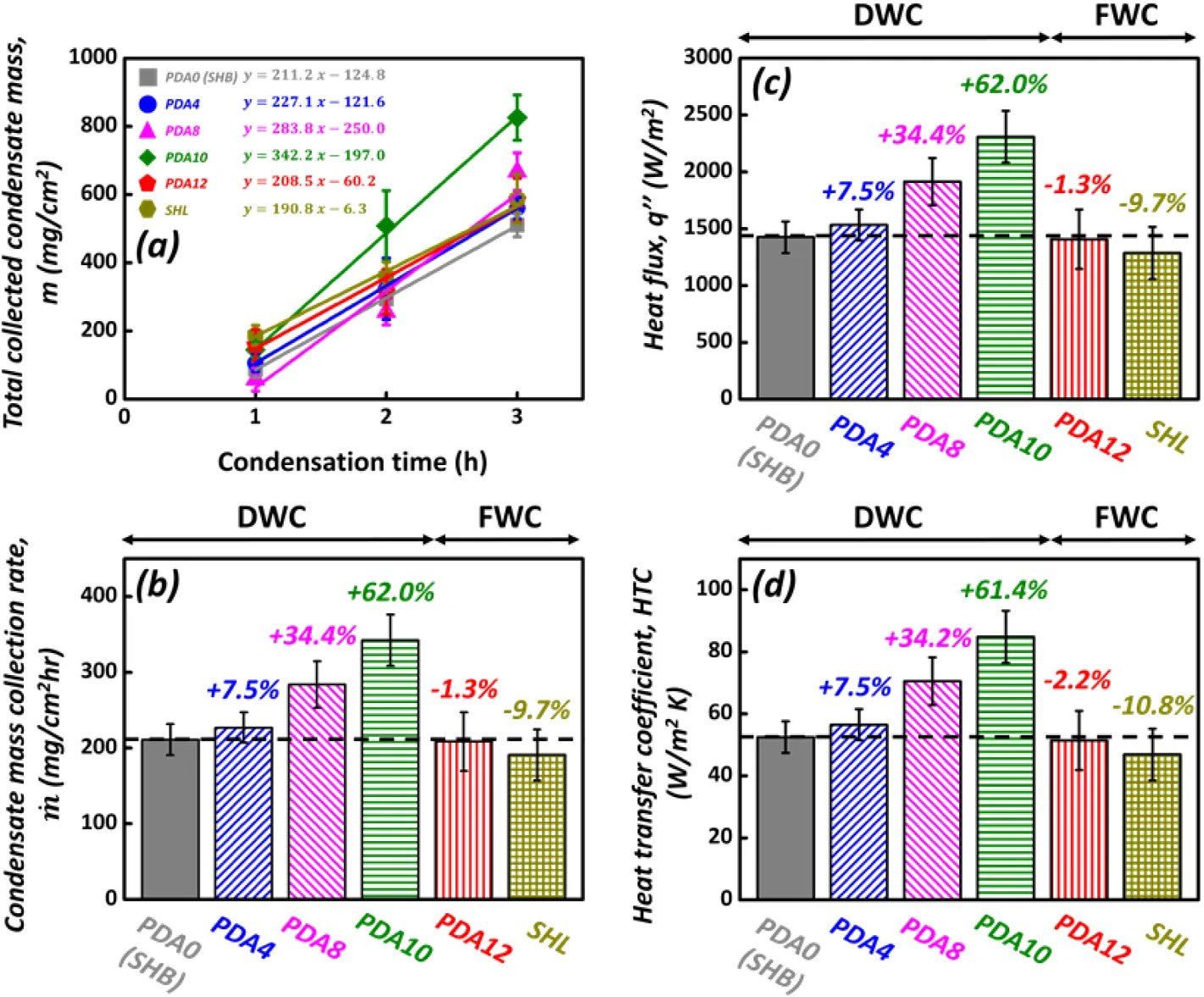
(a) Linear regression of total collected condensate mass per unit area (mg cm^–2^) against condensation time, (b) condensate mass collection rate (*ṁ*), (c) heat flux (q”), and (d) heat transfer coefficient (HTC) of the PDA-modified surfaces.

In comparison, Zhang et al.^35^ reported an inkjet-printed superhydrophilic–superhydrophobic micropatterned surface with a pattern size of approximately 500 μm and a spacing of about 1000 μm for water harvesting. Under an ambient temperature of 22 °C, a relative humidity of 90–95%, and a substrate temperature of approximately 4 °C, the reported water collection rate was approximately 61.8 mg cm^–2^ h^–1^. The water collection rate of PDA10 in this study was approximately 5.5 times that reported by Zhang et al.^35^ Although the experimental conditions were not identical, both studies were conducted under high-humidity and cooled-surface conditions. This comparison further highlights the high water-collection performance of PDA10 without the need for regularly patterned hydrophilic domains.

In contrast, the PDA12 and SHL substrates exhibited FWC. Excessive surface hydrophilicity made the condensed droplets difficult to remove, leaving a water film adhered to the surface. This film continuously covered the surface, suppressing effective surface renewal and negatively affecting condensation. As a result, the condensate mass collection rate of PDA12 decreased by 1.3% relative to PDA0­(SHB), whereas that of SHL decreased by 9.7%. By adjusting surface hydrophilicity, nucleation was enhanced with minimal compromise in droplet removal efficiency, thereby increasing the water collection rate.^50^ An ideal high-efficiency water-harvesting surface must simultaneously promote droplet nucleation and enable efficient droplet removal. While enhanced surface hydrophilicity promotes droplet nucleation, efficient droplet removal requires sufficiently low surface adhesion. To achieve optimal water-harvesting performance, it is essential to strike an appropriate balance between enhancing nucleation and improving removal efficiency. By precisely tuning surface wettability, condensation behavior, and the dynamic removal of droplets, overall water-harvesting efficiency can be effectively optimized.^30,51^

In the heat transfer analysis, accurately determining heat transfer performance was challenging due to the samples’ surface heterogeneity and high roughness. To simplify the analysis of heat transfer performance, the heat flux (*q*”) and the heat transfer coefficient (HTC) were defined as follows:

1 $$q^{\prime \prime }=ṁ\times h_{fg}$$

2 $$\mathrm{HTC}=q^{\prime \prime }/\Delta T=\left(ṁ\times h_{fg}\right)/\Delta T)$$

where the heat flux (W m^–2^) is denoted by *q”*, with *h_fg_* as the latent heat of vaporization (J kg^–1^), *ṁ* as the water collection rate (mg cm^–2^hr^–1^). HTC represents the heat-transfer coefficient (W m^–2^ K^–1^), and Δ*T* is the temperature difference between the saturated temperature (*T*_saturated_) and the surface temperature (*T*_surface_). *T*_surface_ was calculated by averaging the temperatures measured at three positions (upper, middle, and lower) along the y direction at the center of the sample in the x direction, as shown in Figure S14. The experimental data of *T*_saturated_ and *T*_surface_ for each sample are summarized in Table S2.

Figure 11­(c) and 11­(d) present the heat flux (q″) and heat transfer coefficient (HTC), respectively, for the SHB, PDA-modified, and SHL substrates. The HTC of PDA4, PDA8, and PDA10 increased by 7.5%, 34.2%, and 61.4%, respectively, compared to PDA0­(SHB). These results indicate that, when DWC is maintained, appropriate PDA modification can enhance droplet nucleation while retaining effective droplet removal. During condensation, droplet growth and coalescence induce droplet displacement, facilitating droplet removal and continuous surface renewal, thereby improving heat transfer performance. However, excessive PDA modification and increasing surface hydrophilicity make it more difficult for condensed droplets to detach from the surface. The retained liquid film covers the surface, suppressing effective surface renewal and negatively affecting heat transfer. For the PDA12 and SHL substrates, which exhibited FWC, the HTC decreased by 2.2% and 10.8%, respectively, compared with PDA0­(SHB). These results highlight the importance of achieving an appropriate balance between enhanced droplet nucleation and effective droplet removal to obtain superior condensation heat transfer performance.

## Conclusions

In this study, the surface wettability of PDA-modified surfaces was systematically regulated by controlling the PDA reaction time on the PDA0 (SHB) substrate. The PDA-modified surfaces became increasingly hydrophilic as the PDA reaction time increased. The Cassie-to-Wenzel transition of sessile droplets occurred at a PDA reaction time of 9 min. In contrast, the transition from DWC to FWC during condensation occurred at 11 min, indicating that the sessile-droplet wetting transition and the condensation-mode transition did not occur simultaneously.

Moreover, increasing surface hydrophilicity increased nucleation density but also raised the droplet departure diameter. Under DWC conditions, the water-harvesting flux increased with PDA reaction time because enhanced nucleation remained balanced with effective droplet removal. Among the investigated surfaces, PDA10 achieved the optimal balance between enhanced nucleation and favorable droplet mobility. Consequently, PDA10 exhibited a 62.0% higher condensate mass collection rate and a 61.4% higher heat transfer coefficient than PDA0 (SHB), demonstrating its optimal condensation performance.

These findings demonstrate that regulating the PDA modification time can control the boundaries of the asynchronous wetting and condensation transition, thereby identifying the optimal surface condition that enhances droplet nucleation while maintaining effective droplet removal. This strategy offers a practical design principle for optimizing PDA-modified condensation surfaces for water-harvesting and heat-transfer applications.

## Supplementary Material



















## Notes

The SEM images of spherical silica nanoparticles and the particle size distribution; ACA, RCA, and SA of SiO2 nanoparticle-textured surfaces treated with different OTS reaction times; schematic setup of contact-angle measurement; schematic setup of horizontal condensation; schematic setup of vertical condensation; the SEM images of surface characterization on the PDA0­(SHB), PDA12, and PDA60; AFM height and phase images of the PDA-modified surfaces PDA0, PDA10, and PDA12; SEM images and N elemental mapping of the PDA-modified surfaces PDA4, PDA10, and PDA60; C 1s and O 1s high-resolution XPS spectra of the surfaces: PDA0, PDA4, PDA10, and PDA60; ACA, RCA, and SA of a sessile water droplet on FTS-modified superhydrophobic surfaces at different PDA reaction times; illustration of the OM images during horizontal condensation on the surfaces: PDA0 (SHB), PDA4, PDA8, PDA10, and PDA12, taken at 0, 10, 15, 20, 30, 40, and 50 s; analysis of droplet motion before detachment on the PDA10 substrate; sliding angles of droplets and optical images corresponding to the sliding-angle measurements at different volumes on the PDA10 substrate; comparison of the ACA and RCA of surfaces before and after condensation experiments; C 1s, O 1s, and N 1s high-resolution XPS spectra of the PDA10 surface after 24 h of continuous vertical condensation and 1 h of ultrasonication; schematic illustration of the surface temperature measurement positions for determining *T_surface_* (= *T_surface,avg_*); surface wettability and dynamic contact angle for a water droplet on PDA0­(SHB) and PDA-modified surfaces; water-harvesting efficiency and heat-transfer performance of PDA0­(SHB) and PDA-modified surfaces during vertical condensation (PDF)
A 1 h vertical condensation process on PDA0 (Video S1) (MP4)
A 1 h vertical condensation process on PDA4 (Video S2) (MP4)
A 1 h vertical condensation process on PDA8 (Video S3) (MP4)
A 1 h vertical condensation process on PDA10 (Video S4) (MP4)
A 1 h vertical condensation process on PDA12 (Video S5) (MP4)
A 1 h vertical condensation process on SHL (Video S6) (MP4)
The jumping behavior induced by multidroplet coalescence results in droplet detachment from the PDA0 (SHB) substrate (Video S7) (MP4)
The jumping behavior induced by multidroplet coalescence causes droplets to leap over neighboring droplets and collide with one another on the PDA0­(SHB) substrate (Video S8) (MP4)
