# Supplementary material for: Wettability-Tuned Polydopamine-Modified Surfaces for Enhanced Dropwise Condensation and Water Harvesting
Source: Langmuir. 2026 Oct 15;42(42):31351–62. doi: 10.1021/acs.langmuir.6c04242 (PMC13718611; doi:10.1021/acs.langmuir.6c04242)
Supplement: Supplementary file 1 [file la6c04242_si_001.pdf]

Supporting Information

Wettability-Tuned Polydopamine-Modified Surfaces for Enhanced Dropwise Condensation and Water Harvesting

Nai-Ci Zheng[+], Bo-Yan Huang[+], and Li-Jen Chen*

Department of Chemical Engineering, National Taiwan University, Taipei 10617, Taiwan

[+]Nai-Ci Zheng and Bo-Yan Huang contributed equally to this work

* Corresponding author. E-mail address: ljchen@ntu.edu.tw

Supplementary Figures:

Figure S1. The SEM images of spherical silica nanoparticles and the particle size distribution.

Figure S2. The ACA, RCA, and SA of SiO_2 nanoparticle-textured surfaces treated with different OTS reaction times.

Figure S3. Schematic setup of contact-angle measurement: (A) light source; (B) pinhole lens; (C) plano-convex lens; (D) syringe; (E) prepared surface; (F) syringe pump; (G) CCD camera; (H) personal computer with control system software..

Figure S4. Schematic setup of horizontal condensation: (A) personal computer with control system software; (B) CCD camera; (C) optical microscopy; (D) prepared sample; (E) liquid nitrogen container and condensation system controller.

.**Figure S5.** Schematic setup of vertical condensation. (A) CCD camera; (B) extra light source; (C) fans; (D) liquid nitrogen container; (E) condensation system controller; (F) surrounding temperature and humidity sensor; (G) surrounding temperature controllers; (H) surrounding humidity controllers; (I) cooling plate; (J) water container; (K) humidifier; (L) personal computer with control system software.

Figure S6. The SEM images of surface characterization on the (a) PDA0(SHB), (b) PDA12, and (c)

PDA60.

Figure S7. (a) AFM height (first row) and corresponding phase (second row) images of the PDA-modified surfaces PDA0, PDA10, and PDA12, and (b) SEM images (first row) and corresponding N elemental mapping (second row) of the PDA-modified surfaces PDA4, PDA10, and PDA60.

Figure S8. (a) C 1s and (b) O 1s high-resolution XPS spectra of the surfaces: PDA0, PDA4, PDA10, and PDA60.

Figure S9. The ACA, RCA, and SA of a sessile water droplet on FTS-modified superhydrophobic surfaces at different PDA reaction times.

Figure S10. Illustration of the OM images during horizontal condensation on surfaces: (a) PDA0 (SHB), (b) PDA4, (c) PDA8, (d) PDA10, and (e) PDA12 taken at 0, 10, 15, 20, 30, 40, and 50 seconds.

Figure S11. (a) The analysis of droplet motion before detachment on the substrate of PDA10. The orange and blue circles represent the diameters of the droplet used in the sliding angle measurement (D1, 2498 μm) and the natural departure droplet (D2, 4890 μm), respectively. (b) The sliding angle of droplets and (c) Optical images corresponding to the sliding-angle measurements shown in (b) with different volumes on the PDA10 substrate.

Figure S12. Comparison of the ACA and RCA of surfaces before and after condensation experiments. The red square and blue triangle symbols represent, respectively, the ACA and RCA of surfaces as-prepared before condensation. The green square and gray triangle symbols represent, respectively, the ACA and RCA after condensation experiments. The open and filled symbols represent the Cassie state and Wenzel state, respectively.

Figure S13. The C1s, O1s, and N1s high-resolution XPS spectra of the PDA10 surface after (a) 24 h of continuous vertical condensation and (b) 1 h of ultrasonication.

Figure S14. Schematic illustration of the surface temperature measurement positions for determining

$T_{surface}(= T_{surface,avg})$.

Supplementary Tables:

Table S1. Surface wettability and dynamic contact angle for a water droplet on PDA0(SHB) and PDA-modified surfaces.

Table S2 The water harvesting efficiency and heat transfer performance of PDA0(SHB) and PDA-modified surfaces during vertical condensation.

Supplementary Videos:

Video S1. A one-hour vertical condensation process on the PDA0 (SHB).

Video S2. A one-hour vertical condensation process on the PDA4.

Video S3. A one-hour vertical condensation process on the PDA8.

Video S4. A one-hour vertical condensation process on the PDA10.

Video S5. A one-hour vertical condensation process on the PDA12.

Video S6. A one-hour vertical condensation process on the SHL.

Video S7. The jumping behavior induced by multi-droplet coalescence results in droplet detachment from the PDA0(SHB) substrate.

Video S8. The jumping behavior induced by multi-droplet coalescence causes droplets to leap over neighboring droplets and collide with one another on the PDA0(SHB) substrate.

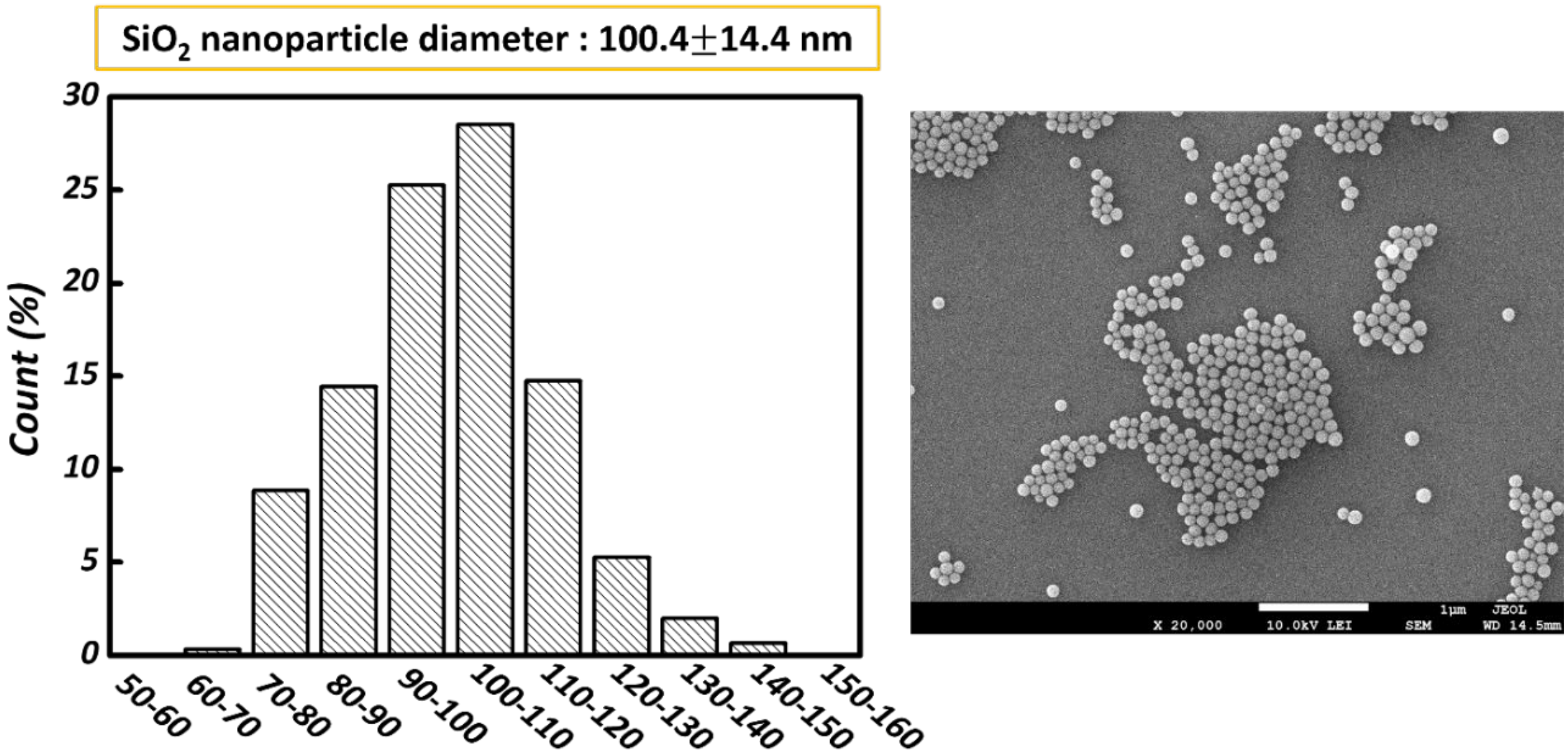

Figure S1. The SEM images of spherical silica nanoparticles and the particle size distribution.

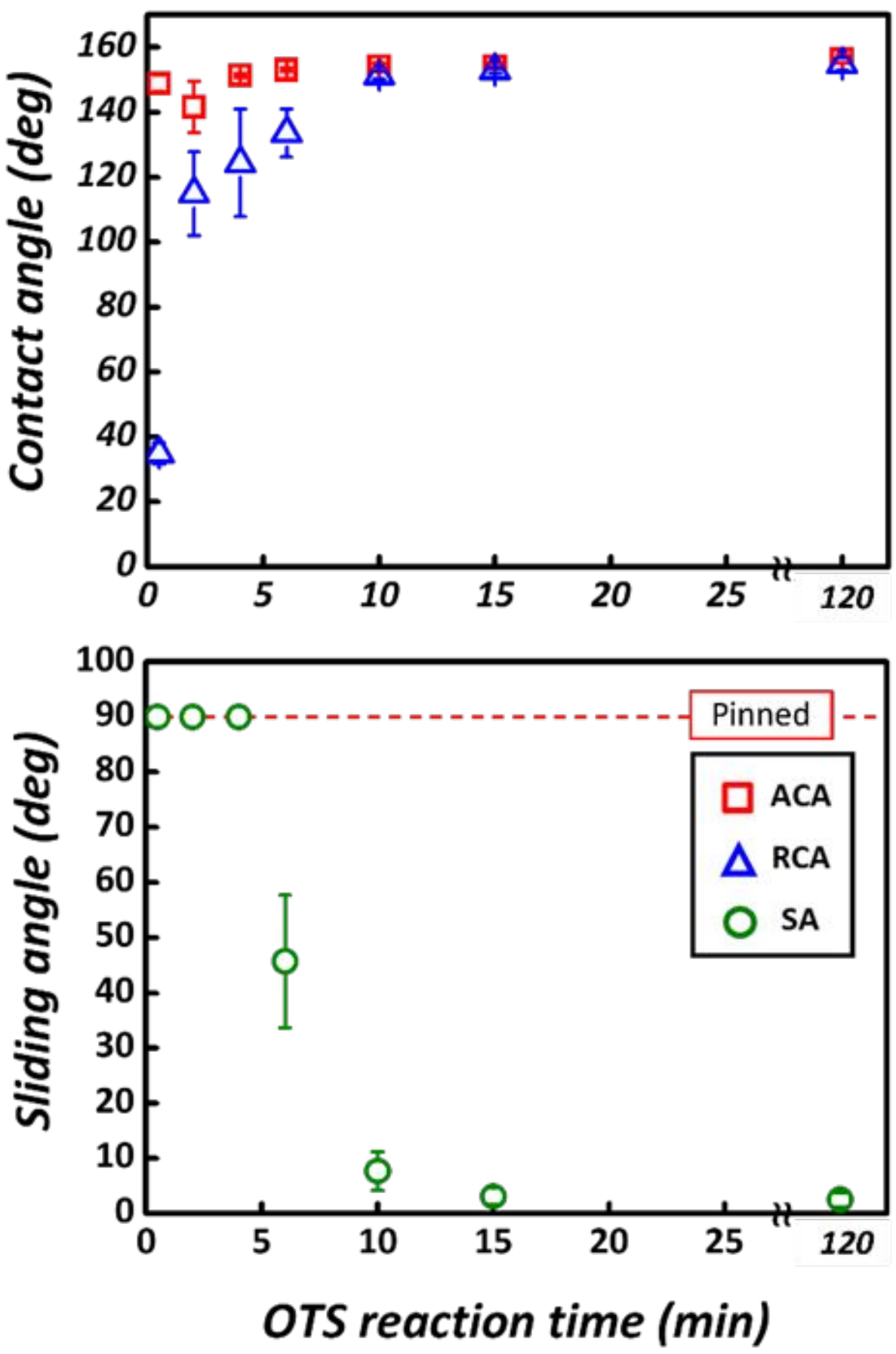

Figure S2. The ACA, RCA, and SA of SiO_2 nanoparticle-textured surfaces treated with different OTS reaction times.

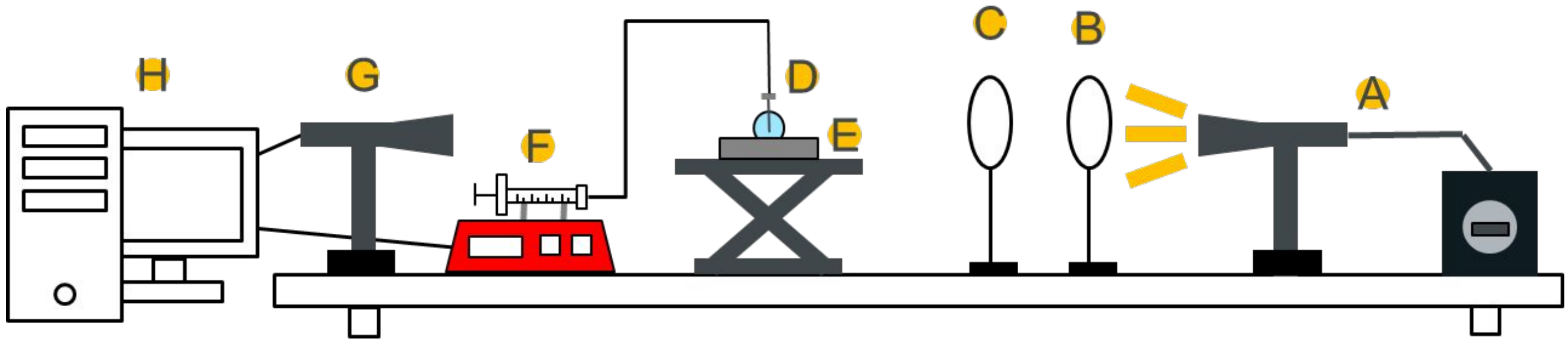

Figure S3. Schematic setup of contact-angle measurement: (A) light source; (B) pinhole lens; (C) plano-convex lens; (D) syringe; (E) prepared surface; (F) syringe pump; (G) CCD camera; (H) personal computer with control system software.

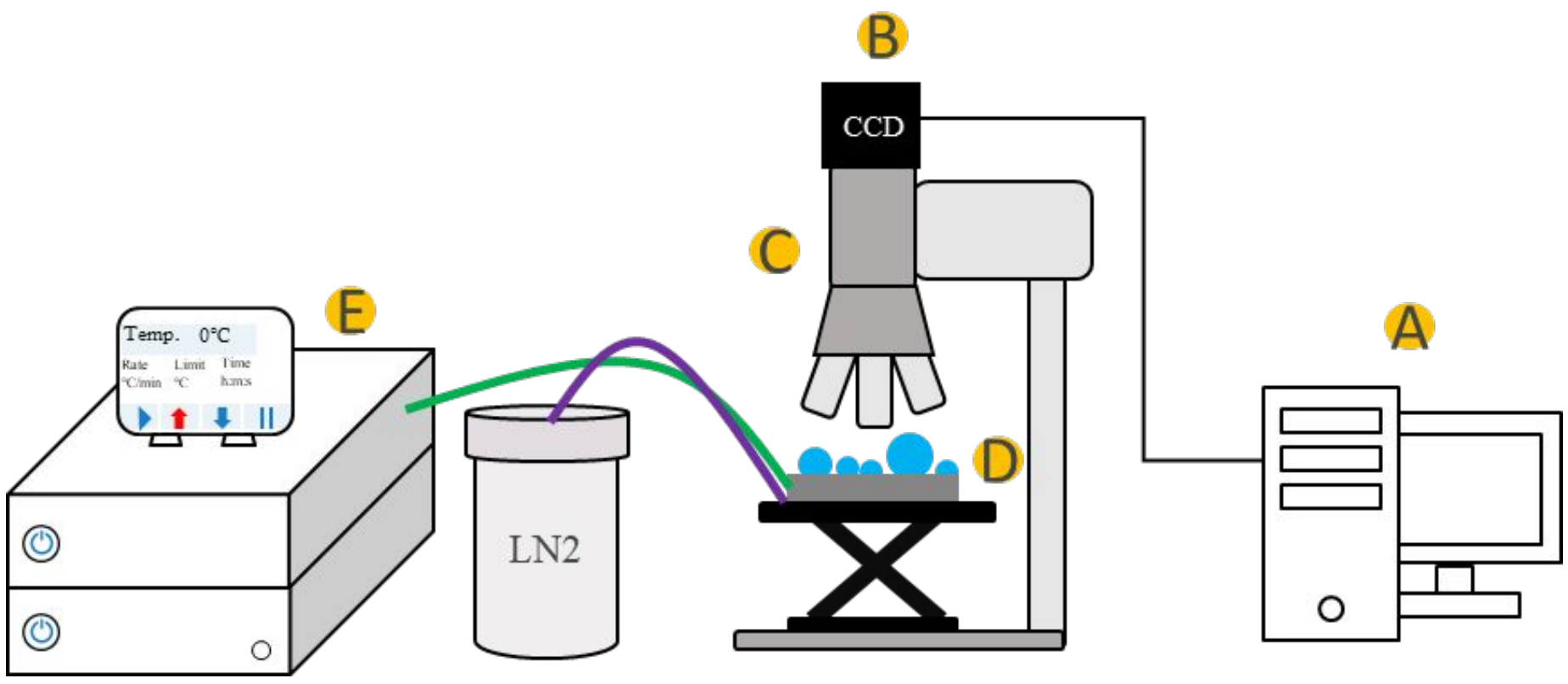

Figure S4. Schematic setup of horizontal condensation: (A) personal computer with control system software; (B) CCD camera; (C) optical microscopy; (D) prepared sample; (E) liquid nitrogen container and condensation system controller.

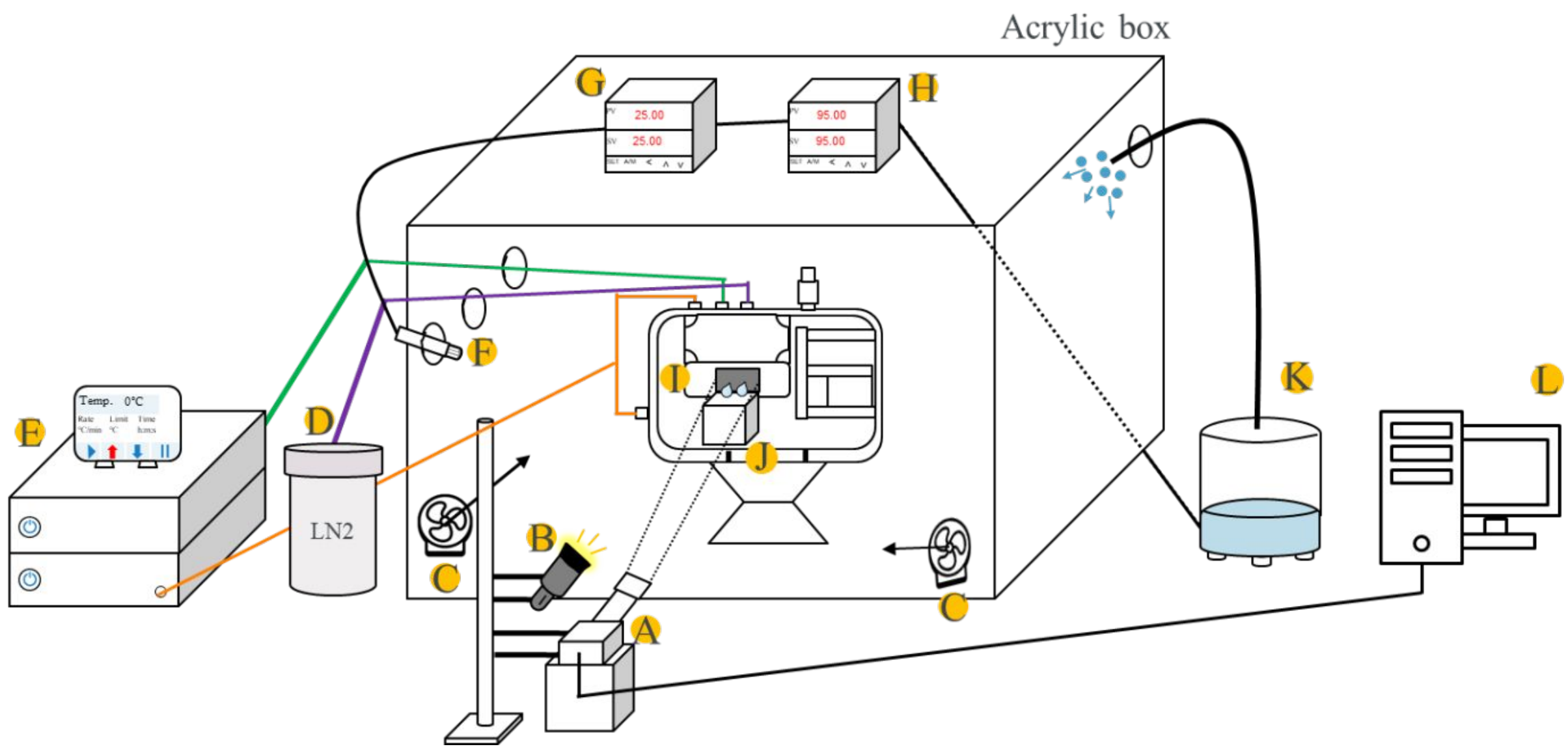

Figure S5. Schematic setup of vertical condensation. (A) CCD camera; (B) extra light source; (C) fans; (D) liquid nitrogen container; (E) condensation system controller; (F) surrounding temperature and humidity sensor; (G) surrounding temperature controllers; (H) surrounding humidity controllers; (I) cooling plate; (J) water container; (K) humidifier; (L) personal computer with control system software.

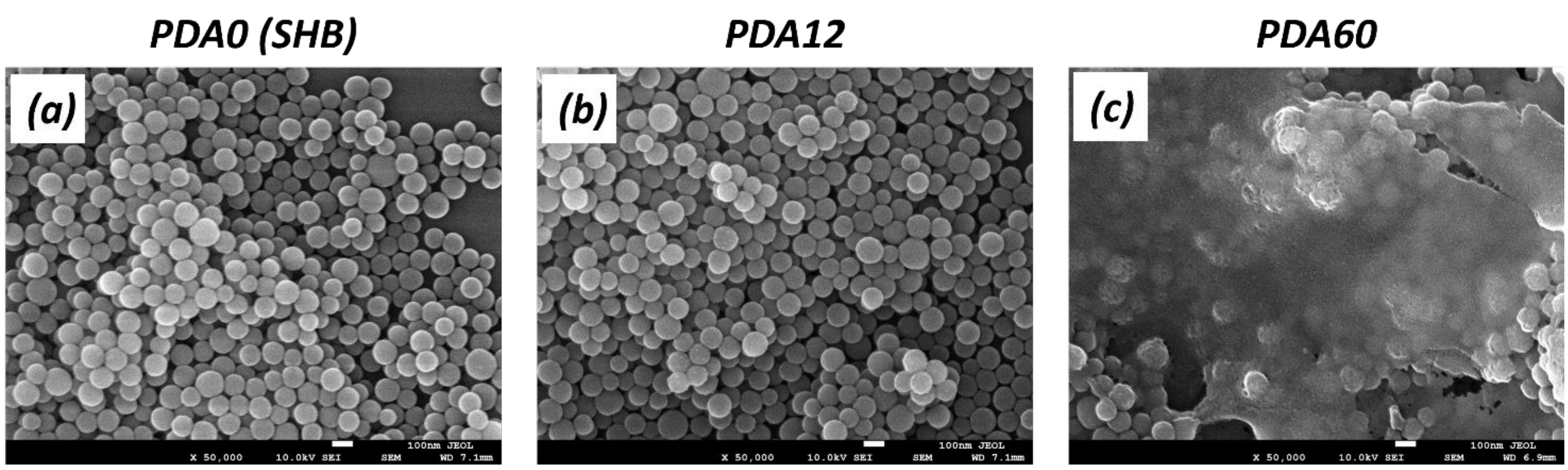

Figure S6. The SEM images of surface characterization on the (a) PDA0(SHB), (b) PDA12, and (c) PDA60.

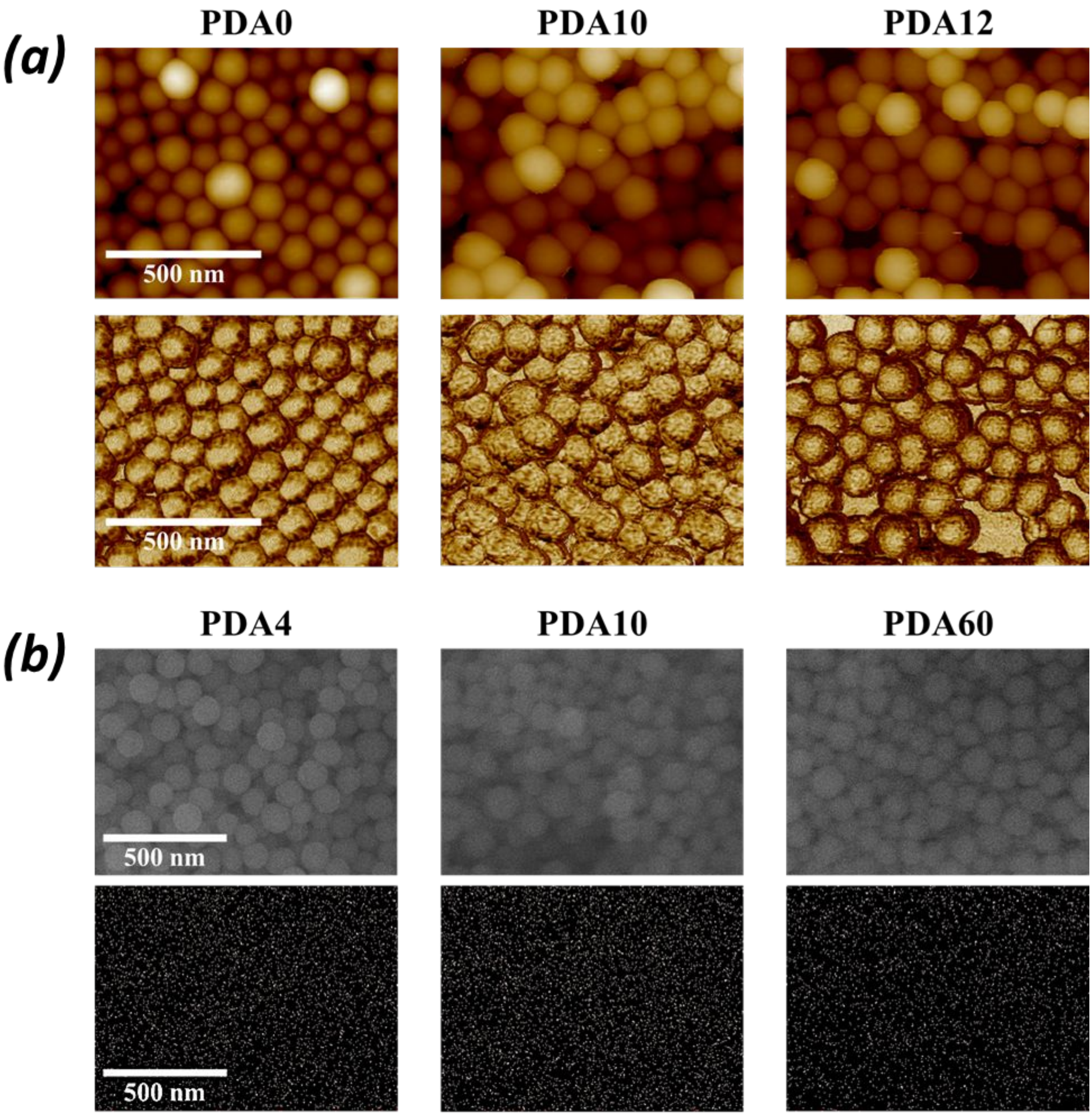

Figure S7. (a) AFM height (first row) and corresponding phase (second row) images of the PDA-modified surfaces PDA0, PDA10, and PDA12, and (b) SEM images (first row) and corresponding N elemental mapping (second row) of the PDA-modified surfaces PDA4, PDA10, and PDA60.

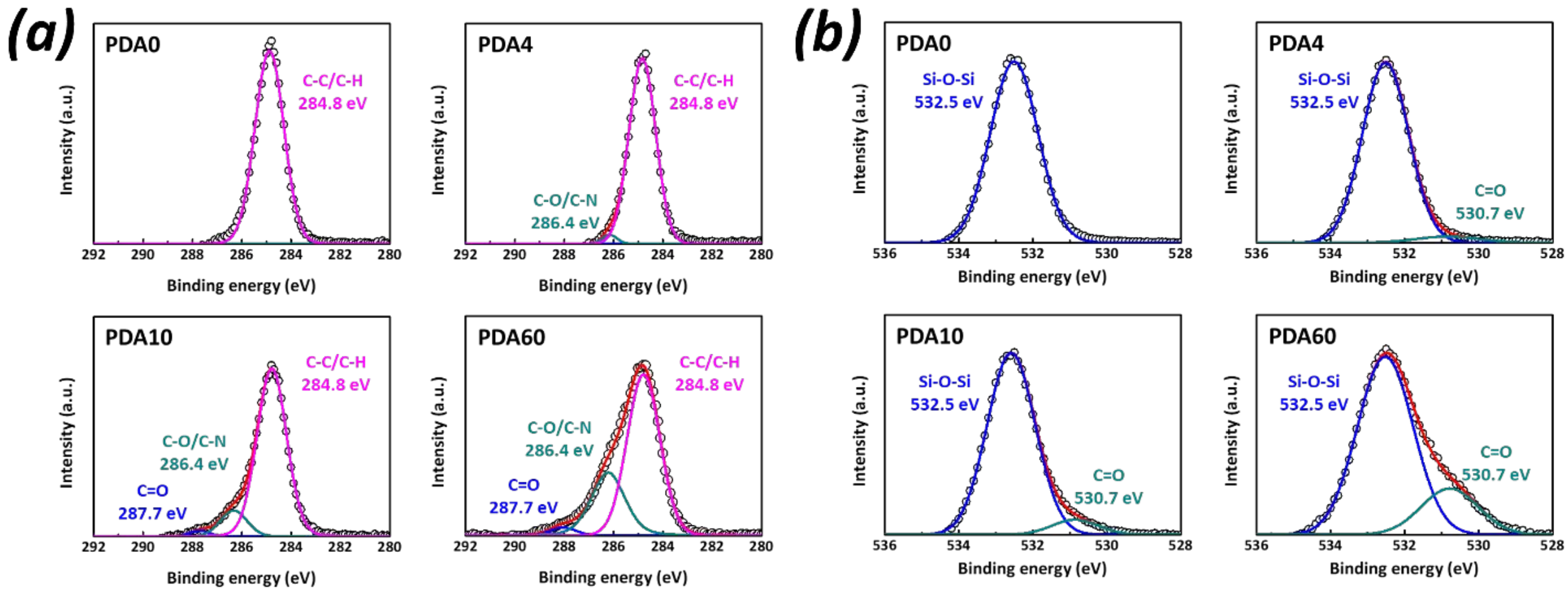

Figure S8. (a) C 1s and (b) O 1s high-resolution XPS spectra of the surfaces: PDA0, PDA4, PDA10, and PDA60.

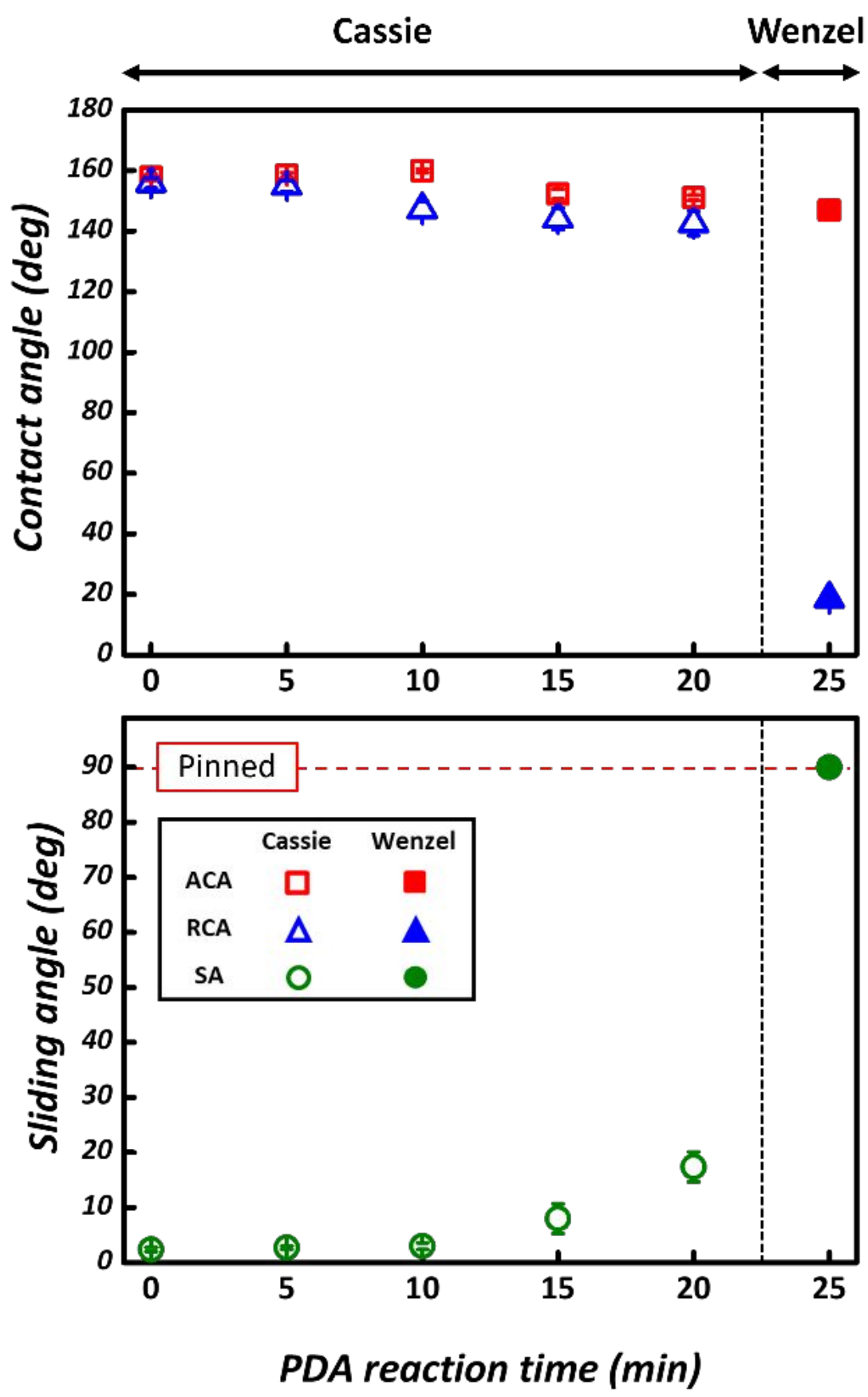

Figure S9. The ACA, RCA, and SA of a sessile water droplet on FTS-modified superhydrophobic surfaces at different PDA reaction times.

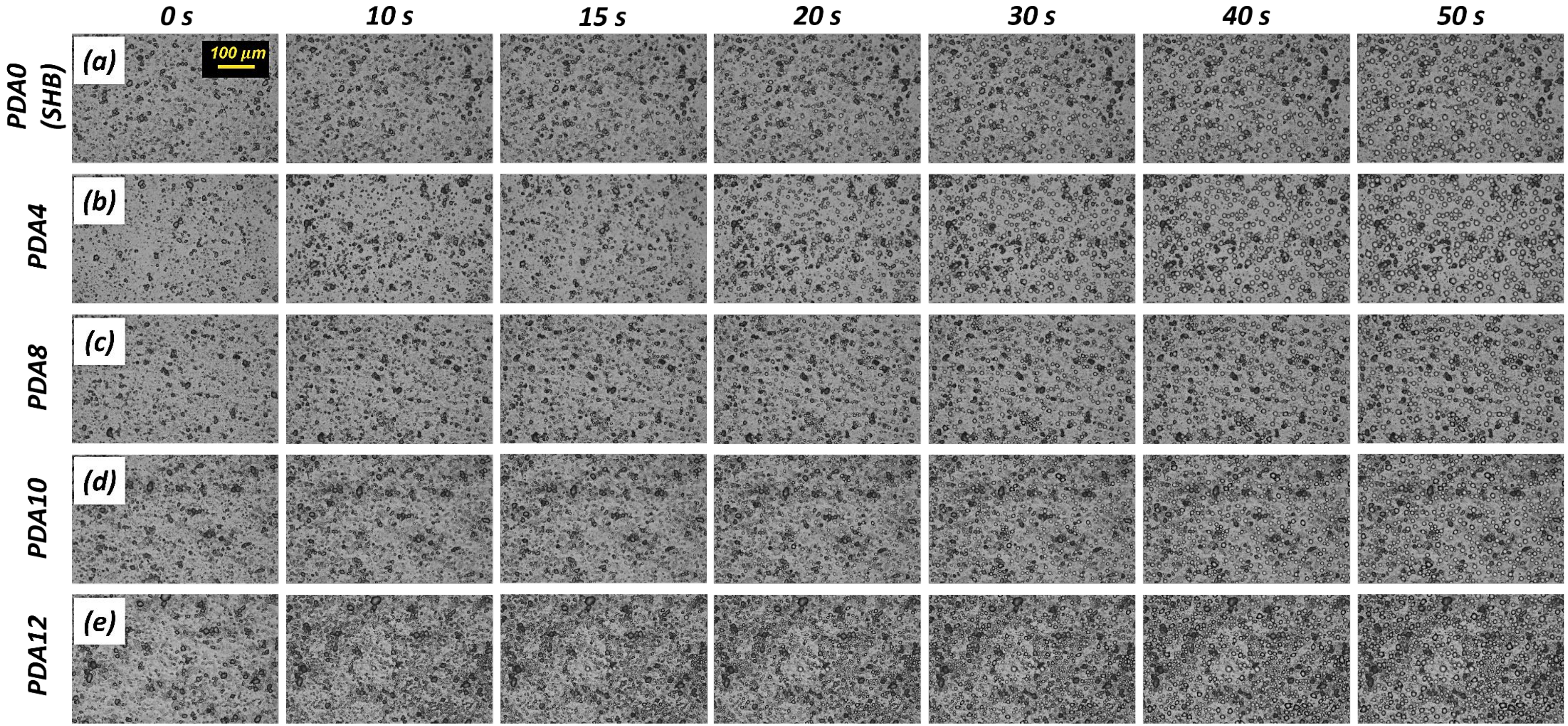

Figure S10. Illustration of the OM images during horizontal condensation on surfaces: (a) PDA0 (SHB), (b) PDA4, (c) PDA8, (d) PDA10, and (e) PDA12 taken at 0, 10, 15, 20, 30, 40, and 50 seconds.

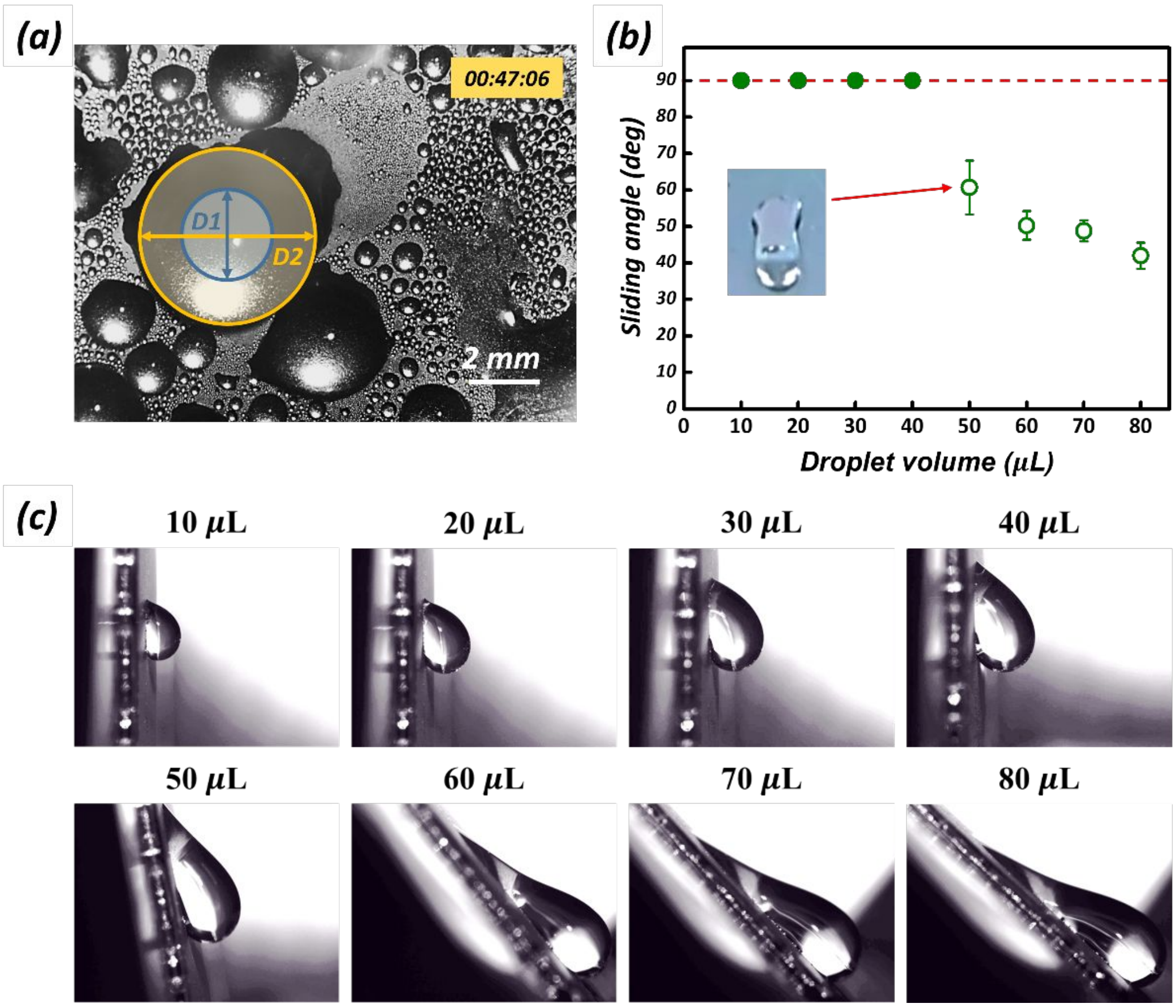

Figure S11. (a) The analysis of droplet motion before detachment on the substrate of PDA10. The orange and blue circles represent the diameters of the droplet used in the sliding angle measurement (D1, 2498 μm) and the natural departure droplet (D2, 4890 μm), respectively. (b) The sliding angle of droplets and (c) Optical images corresponding to the sliding-angle measurements shown in (b) with different volumes on the PDA10 substrate.

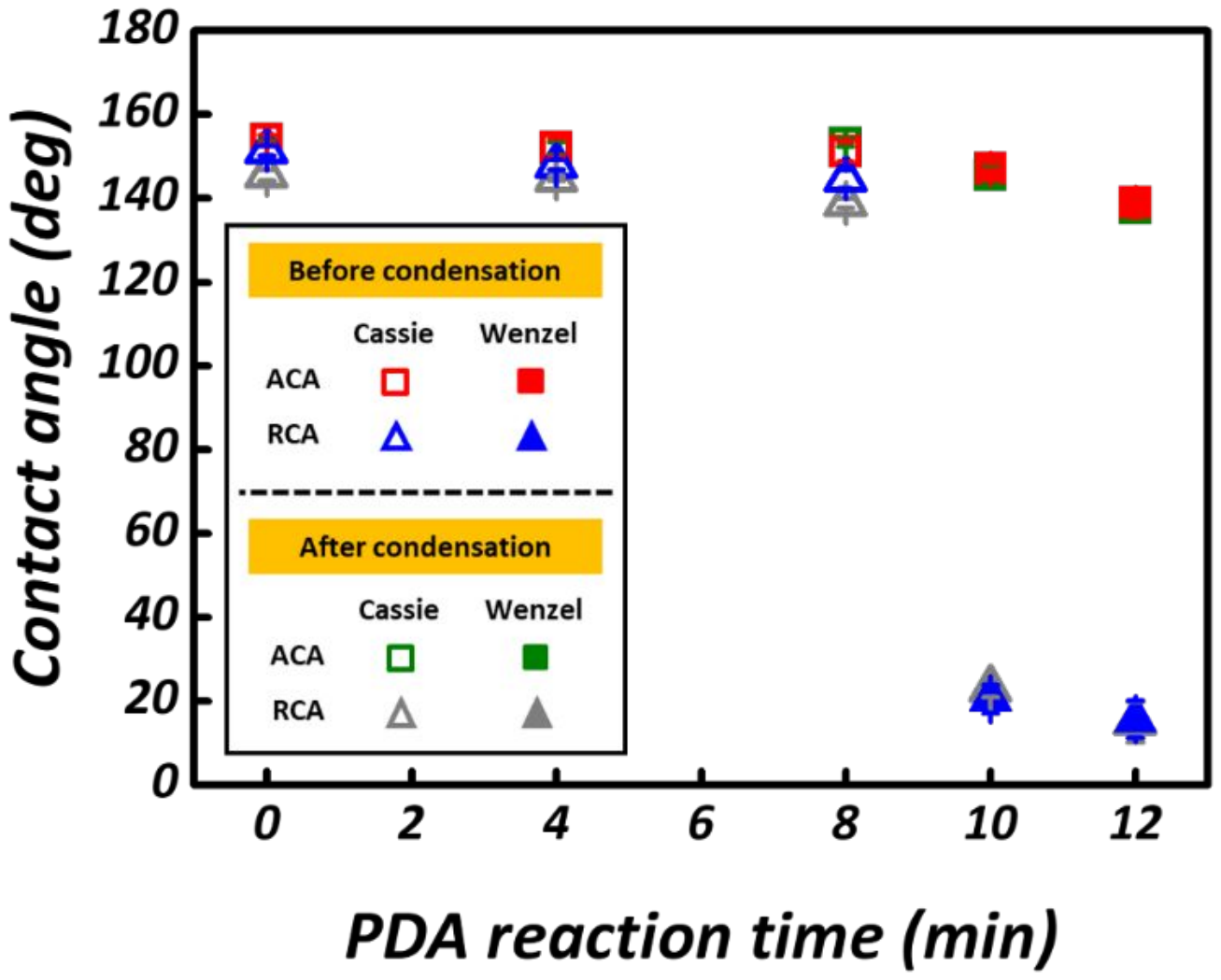

Figure S12. Comparison of the ACA and RCA of surfaces before and after condensation experiments. The red square and blue triangle symbols represent, respectively, the ACA and RCA of surfaces as-prepared before condensation. The green square and gray triangle symbols represent, respectively, the ACA and RCA after condensation experiments. The open and filled symbols represent the Cassie state and Wenzel state, respectively.

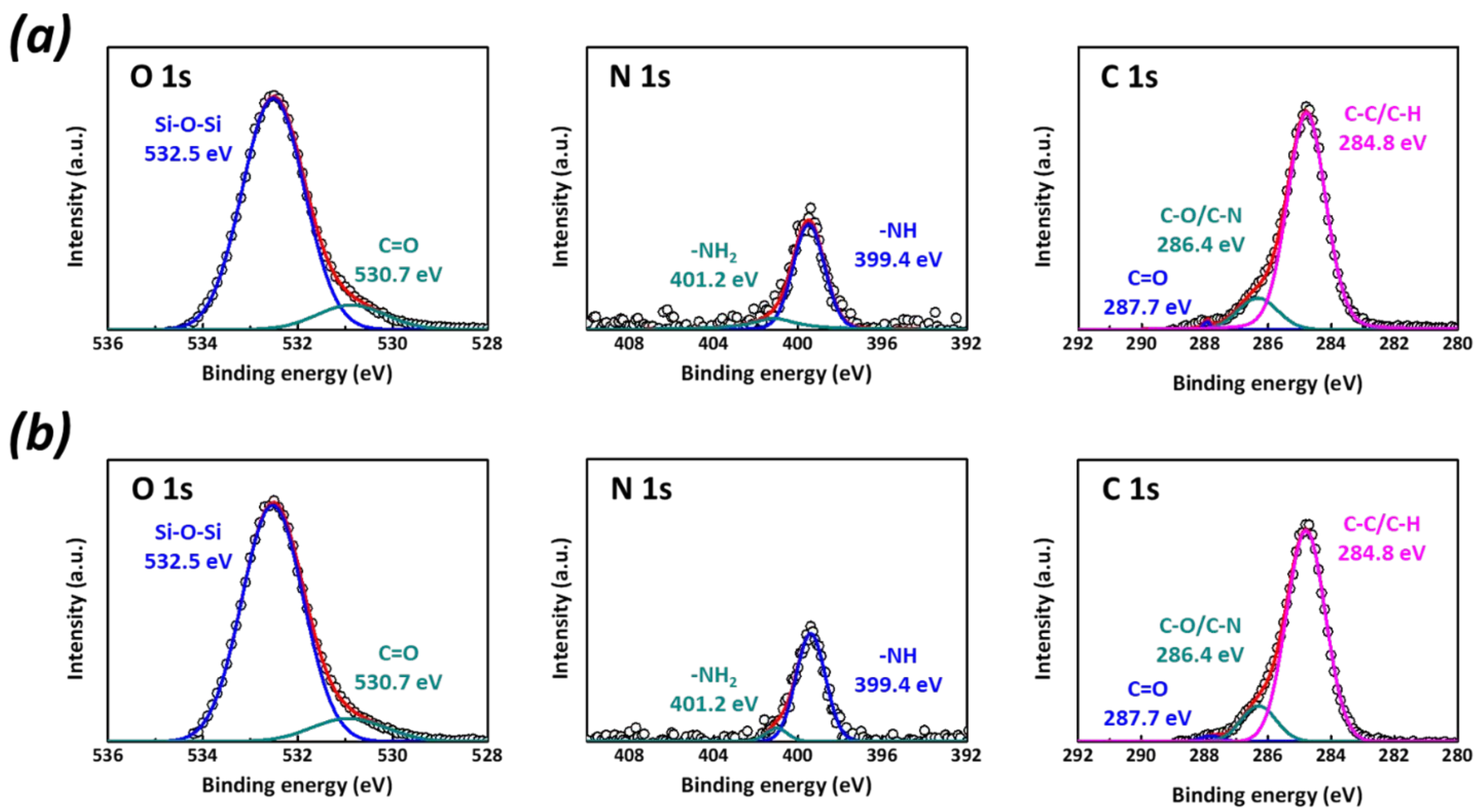

Figure S13. High-resolution C1s, O1s, and N1s XPS spectra of the PDA10 surface after (a) 24 h of continuous vertical condensation and (b) 1 h of ultrasonication.

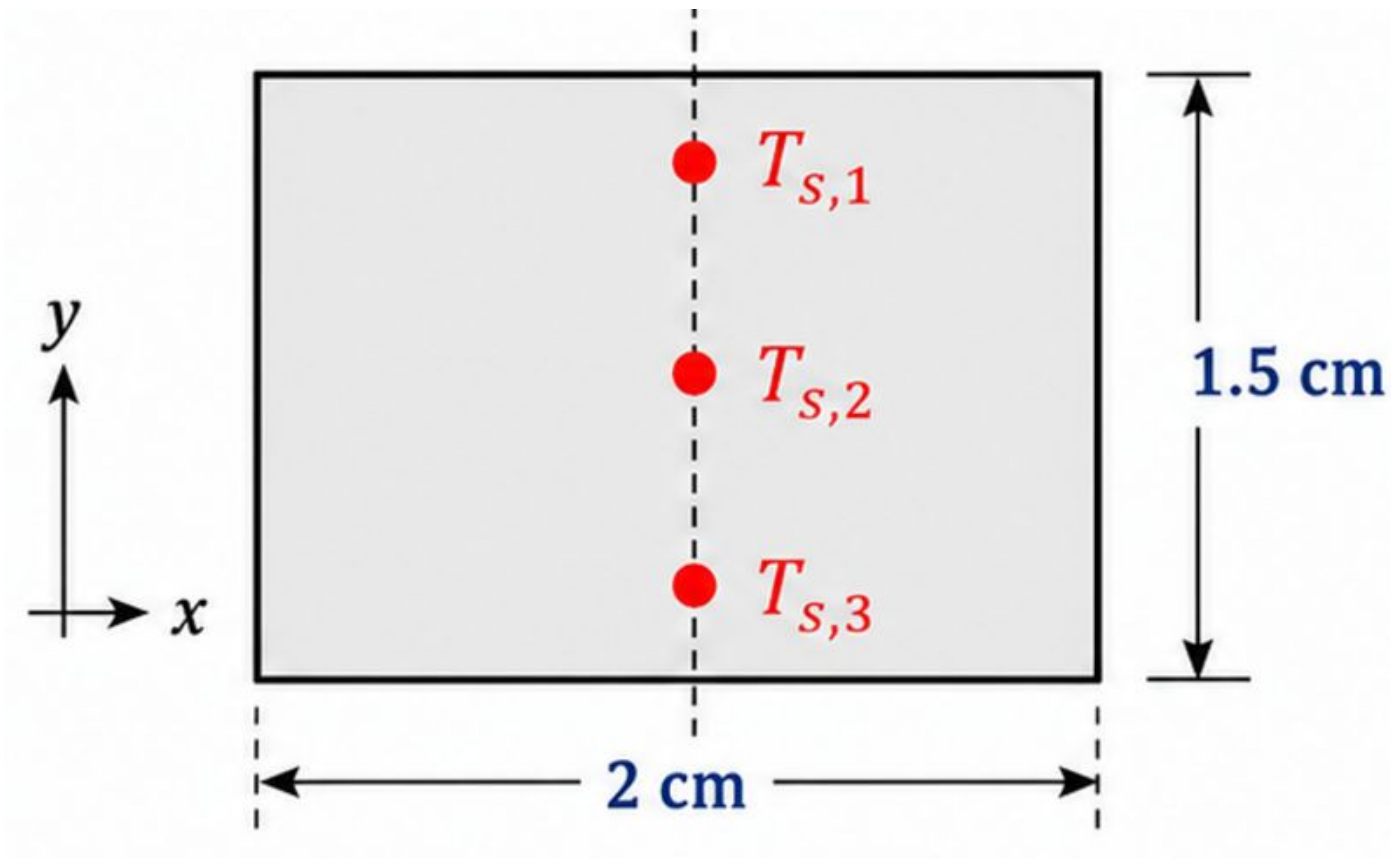

$$T_{surface,avg} = \frac{T_{s,1} + T_{s,2} + T_{s,3}}{3}$$

Figure S14. Schematic illustration of the surface temperature measurement positions for determining $T_{surface}(= T_{surface,avg})$.

Table S1. Surface wettability and dynamic contact angle for a water droplet on PDA0(SHB) and PDA-modified surfaces.

	**PDA0 (SHB)**	**PDA4**	**PDA8**	**PDA10**	**PDA12**
**Surface wettability**	Cassie state	Cassie state	Cassie state	Wenzel state	Wenzel state
**ACA (º)**	154.1 ± 1.0	152.2 ± 2.6	151.1 ± 2.3	147.0 ± 1.5	139.0 ± 3.4
**RCA (º)**	151.3 ± 1.2	147.9 ± 1.1	144.6 ± 2.2	20.5 ± 3.4	15.7 ± 4.5
**SA (º)**	7.7 ± 3.5	8.9 ± 5.2	12.1 ± 4.1	Pinned	Pinned

Table S2. The water harvesting efficiency and heat transfer performance of PDA0(SHB) and PDA-modified surfaces during vertical condensation.

	**PDA0 (SHB)**	**PDA4**	**PDA8**	**PDA10**	**PDA12**	**SHL**
**Condensation mechanism**	Cassie / DWC	Cassie / DWC	Cassie / DWC	Wenzel/ DWC	Wenzel / FWC	Wenzel / FWC
$T_{surface}$ (℃)[a]	2.2 ± 0.1	2.2 ± 0.2	2.1 ± 0.1	2.1 ± 0.1	1.9 ± 0.1	1.8 ± 0.1
$T_{saturated}$ (℃)	29.3					
A_S (cm^2)	2.55 ± 0.08	2.53 ± 0.15	3.02 ± 0.13	2.86 ± 0.06	2.87 ± 0.06	2.77 ± 0.01
$\dot{m}$ (mg cm^{-2} hr^{-1})[b]	211.2 ± 20.6	227.1 ± 20.0	283.8 ± 30.8	342.2 ± 33.7	208.5 ± 38.6	190.8 ± 33.8
**q''(W m^{-2})**	1425.6 ± 139.1	1532.9 ± 135.0	1915.7 ± 207.9	2309.9 ± 227.5	1407.4 ± 260.6	1287.9± 228.2
**HTC (W m^{-2} K^{-1})**	52.5 ± 5.1	56.5 ± 5.0	70.5 ± 7.7	84.8 ± 8.4	51.4 ± 9.5	46.9 ± 8.3

[a] $T_{surface}$ was calculated by averaging the temperatures measured at three positions (upper, middle, and lower) along the y direction at the center of the sample in the x direction

[b] $\dot{m}$ is the slope obtained from a linear regression of the total collected condensate mass (y-axis) versus condensation time (x-axis).
